# Mechanisms underlying a thalamocortical transformation during active tactile sensation

**DOI:** 10.1371/journal.pcbi.1005576

**Published:** 2017-06-07

**Authors:** Diego Adrian Gutnisky, Jianing Yu, Samuel Andrew Hires, Minh-Son To, Michael Ross Bale, Karel Svoboda, David Golomb

**Affiliations:** 1 Janelia Research Campus, HHMI, Ashburn, Virginia, United States of America; 2 Dept. of Biological Sciences, Neurobiology Section, University of Southern California, Los Angeles, California, United States of America; 3 Dept. of Human Physiology and Centre for Neuroscience, Flinders University, South Australia, Australia; 4 Faculty of Life Sciences, University of Manchester, United Kingdom; 5 School of Life Sciences, University of Sussex, United Kingdom; 6 Depts. of Physiology and Cell Biology and Physics and Zlotowski Center for Neuroscience, Faculty of Health Sciences, Ben Gurion University, Be’er-Sheva, Israel; UCL, UNITED KINGDOM

## Abstract

During active somatosensation, neural signals expected from movement of the sensors are suppressed in the cortex, whereas information related to touch is enhanced. This tactile suppression underlies low-noise encoding of relevant tactile features and the brain’s ability to make fine tactile discriminations. Layer (L) 4 excitatory neurons in the barrel cortex, the major target of the somatosensory thalamus (VPM), respond to touch, but have low spike rates and low sensitivity to the movement of whiskers. Most neurons in VPM respond to touch and also show an increase in spike rate with whisker movement. Therefore, signals related to self-movement are suppressed in L4. Fast-spiking (FS) interneurons in L4 show similar dynamics to VPM neurons. Stimulation of halorhodopsin in FS interneurons causes a reduction in FS neuron activity and an increase in L4 excitatory neuron activity. This decrease of activity of L4 FS neurons contradicts the "paradoxical effect" predicted in networks stabilized by inhibition and in strongly-coupled networks. To explain these observations, we constructed a model of the L4 circuit, with connectivity constrained by in vitro measurements. The model explores the various synaptic conductance strengths for which L4 FS neurons actively suppress baseline and movement-related activity in layer 4 excitatory neurons. Feedforward inhibition, in concert with recurrent intracortical circuitry, produces tactile suppression. Synaptic delays in feedforward inhibition allow transmission of temporally brief volleys of activity associated with touch. Our model provides a mechanistic explanation of a behavior-related computation implemented by the thalamocortical circuit.

## Author summary

We study how information is transformed between connected brain areas: the thalamus, the gateway to the cortex, and layer 4 (L4) in cortex, which is the first station to process sensory input from the thalamus. When mice perform an active object localization task with their whiskers, thalamic neurons and inhibitory fast-spiking (FS) interneurons in L4 encode whisker movement and touch, whereas L4 excitatory neurons respond almost exclusively to touch. To explain these observations, we constructed a computational model based on measured circuit parameters. The model reveals that without touch, when thalamic activity varies slowly, strong inhibition from FS neurons prevents activity in L4 excitatory neurons. Brief and strong touch-induced thalamic activity excites both excitatory and FS neurons in L4. FS neurons inhibit excitatory neurons with a delay of approximately 1 ms relative to ascending excitation, allowing L4 excitatory neurons to spike. Our results demonstrate that cortical circuits exploit synaptic delays for fast computations. Similar mechanisms likely also operate for rapid stimuli in the visual and auditory systems.

## Introduction

Thalamocortical circuits represent model systems for multi-area computations [1]. Sensory information enters the cortex through the thalamus. Transformations in thalamocortical circuits have mostly been studied in anesthetized animals with passive sensory stimuli [2–8] or with artificial whisking [9]. In the somatosensory system these studies have revealed subtle differences in receptive field structure across neurons in the thalamocortical circuit [2–4].

However, active sensation in awake animals involves dynamic interactions with the world, such as saccades [10], palpation with the digits of the hand [11], or movements of the whiskers on the face of rodents [12–14]. During active sensation, movement of the sensors produces ‘reafferent’ signals, whereas interactions with the world generate ‘exafferent’ signals. During haptic exploration, movement activates peripheral sensors to produce reafference and touch generates exafference [15–21]. The brain needs to parse these different signals for perception [13]. During active sensation, movement attenuates the transmission of certain sensory signals to the cortex [22–24]. Tactile suppression is thought to enhance perception of salient events that cannot be predicted based on movement. Tactile suppression is an example of adaptive filtering [25, 26], which is critical for low-noise encoding of relevant sensory stimuli. Here we identify the mechanisms of adaptive filtering in the thalamocortical circuit of the mouse whisker system.

Whisker touch and movement are transduced by mechanosensory afferents in the whisker follicle. Information then flows through the trigeminal ganglion, to the brainstem, thalamus (barreloids in the ventral posterior medial thalamic nucleus, VPM) and terminates in the primary somatosensory cortex (vS1). The main target of VPM axons is the Layer 4 (L4) barrels in vS1. The microcircuit of each L4 barrel is mostly contained within the barrel, and the connections between specific cell types within the barrel have been mapped: L4 excitatory neurons and L4 fast-spiking, parvalbumin (PV)-expressing GABAergic interneurons (FS) are connected within type and across types [27, 28]. Apart from neuromodulation, the only known long-range input to L4 originates in VPM [29–31]. VPM excites all L4 neuron types, and L4 FS neurons inhibit the excitatory neurons to implement feedforward inhibition [32–35].

Cell type-specific recordings from VPM, L4 excitatory [36] and L4 FS neurons [37] uncovered a fundamental computation performed by L4 barrels. Neurons in VPM respond to touch, but they also increase their activity during whisker movement (‘whisking’) [17–21]. L4 FS neurons have nearly identical dynamics as VPM neurons. In contrast, L4 excitatory neuron spikes are strongly coupled to touch, but respond only weakly to whisking [36]. L4 microcircuits therefore transmit touch signals and suppress reafferent signals generated by whisking. These observations indicate that the thalamocortical circuit accentuates salient tactile information by suppressing signals related to self-movement.

Multiple observations regarding the L4 circuit remain to be explained [37]. First, the circuit suppresses self-movement signals but transmit touch signals. From a theoretical perspective, this selective filtering violates the linear response expected from theories of strongly coupled cortical networks [38, 39]. Second, whereas the baseline spike rates of VPM and L4 FS neurons are substantial during baseline conditions and more than double during whisking, the spike rate of L4 excitatory neurons is very low at baseline and does not increase during whisking. Third, when an inhibitory opsin (halorhodopsin) is photostimulated in L4 FS neurons, the spike rates of FS neurons decrease and the spike rates of L4 excitatory neurons increase [37]. Although this result is naively expected, it contradicts the "paradoxical effect" predicted from models of neural circuits that are stabilized by inhibition and from models of strongly-coupled networks [40–42]. According to these models, both inhibitory and excitatory neurons should respond with *increased* spike rates at modest levels of inactivation of inhibitory neurons [41].

To understand the mechanisms underlying these experimental observations we constructed and analyzed a conductance-based model of the L4 circuit. The synaptic circuits of L4 of the barrel cortex have been studied in great detail in brain slices [27–29, 43–46]. These measurements allowed us to constrain the numbers of neurons, neuronal properties, the patterns of connectivity, and the average synaptic strengths in the model. We set parameter values near values extracted from these measurements. This "reference set of parameters" was adjusted over a restricted range, such that the model circuit displayed dynamics similar to the actual L4 circuit. We then varied parameters to explore their roles in controlling system dynamics. We studied how the model network responds to thalamic input at baseline, during whisker movement and during touch. We used the model to disentangle the roles of feedforward synaptic connections from the thalamus and recurrent intracortical connections in shaping L4 dynamics. The model revealed an important role for synaptic delays, fast synaptic kinetics, and inhibitory and excitatory conductance strengths in shaping the L4 responses during behavior.

## Results

### Thalamocortical transformation of tactile information

**Fig 1:**
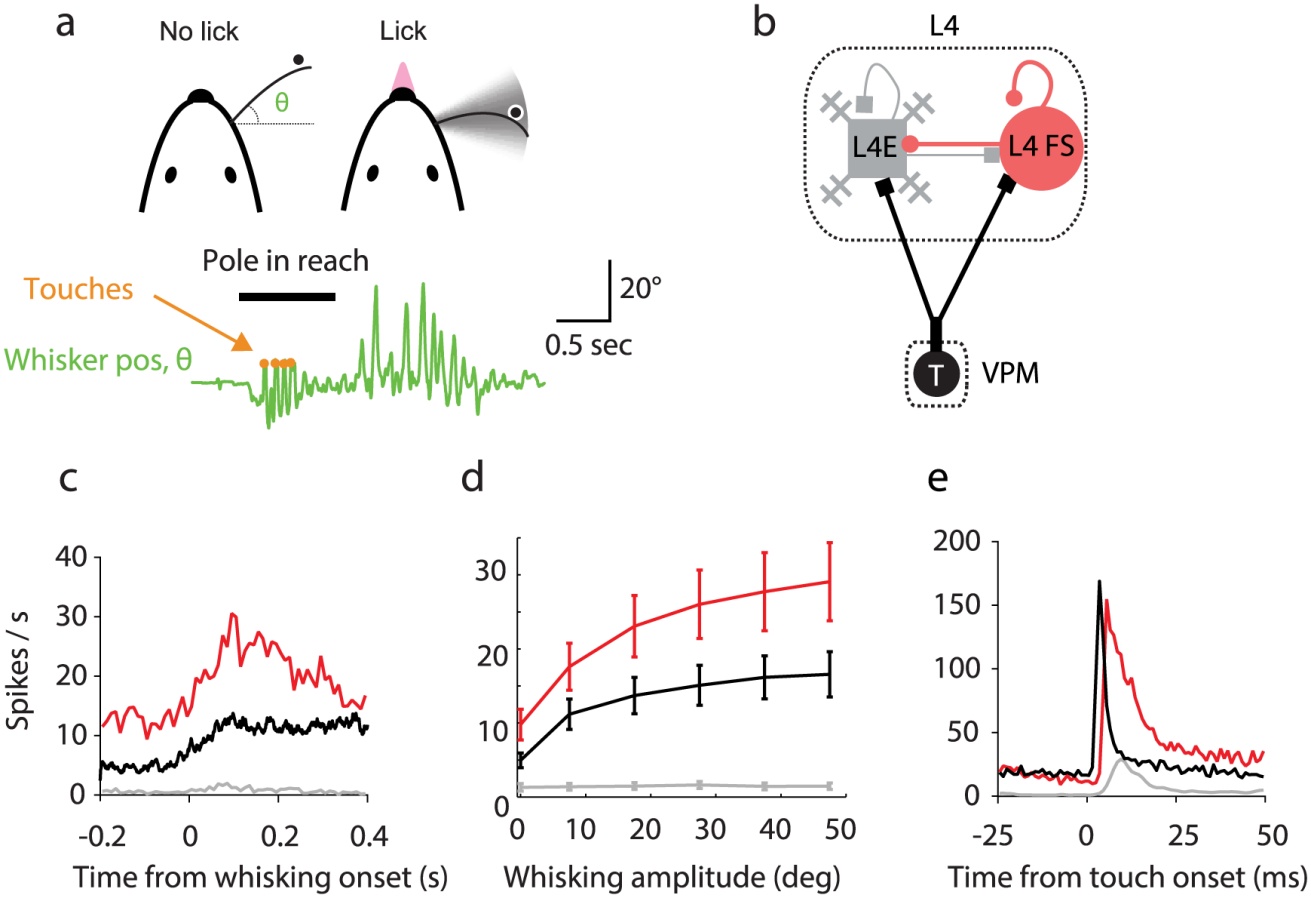
Summary of experimental results in the object-localization task [37]. **(a)** Top, schematic illustrates measurement of whisker position (azimuthal angle *θ*), instances of touch and an example trace of whisker position. Protraction corresponds to positive changes in *θ*. **(b)** Schematics of the thalamocortical circuit and relevant cell-types. **(c)** Spike rate aligned to transitions from non-whisking to whisking (adapted from panel 5e in [37]). **(d)** Average spike rate as a function of whisking amplitude. **(e)** Average population response aligned to touch (adapted from panel 5c in [37]). Data and figures corresponding to previously reported datasets [36, 37].

We first summarize recordings made in VPM and in L4 from excitatory and FS GABAergic interneurons in mice performing an object location discrimination behavior (Fig 1a and 1b). Head-restrained mice had to localize a vertical pole with their whiskers for a water reward [47, 48]. Whisker movement and touch were tracked on millisecond time scales with high-speed videography [49, 50] (Fig 1a). Recordings were made with extracellular silicon probe recordings in VPM [37], and loose-seal cell-attached and whole cell recordings in L4 [36, 37, 51].

**Fig 2:**
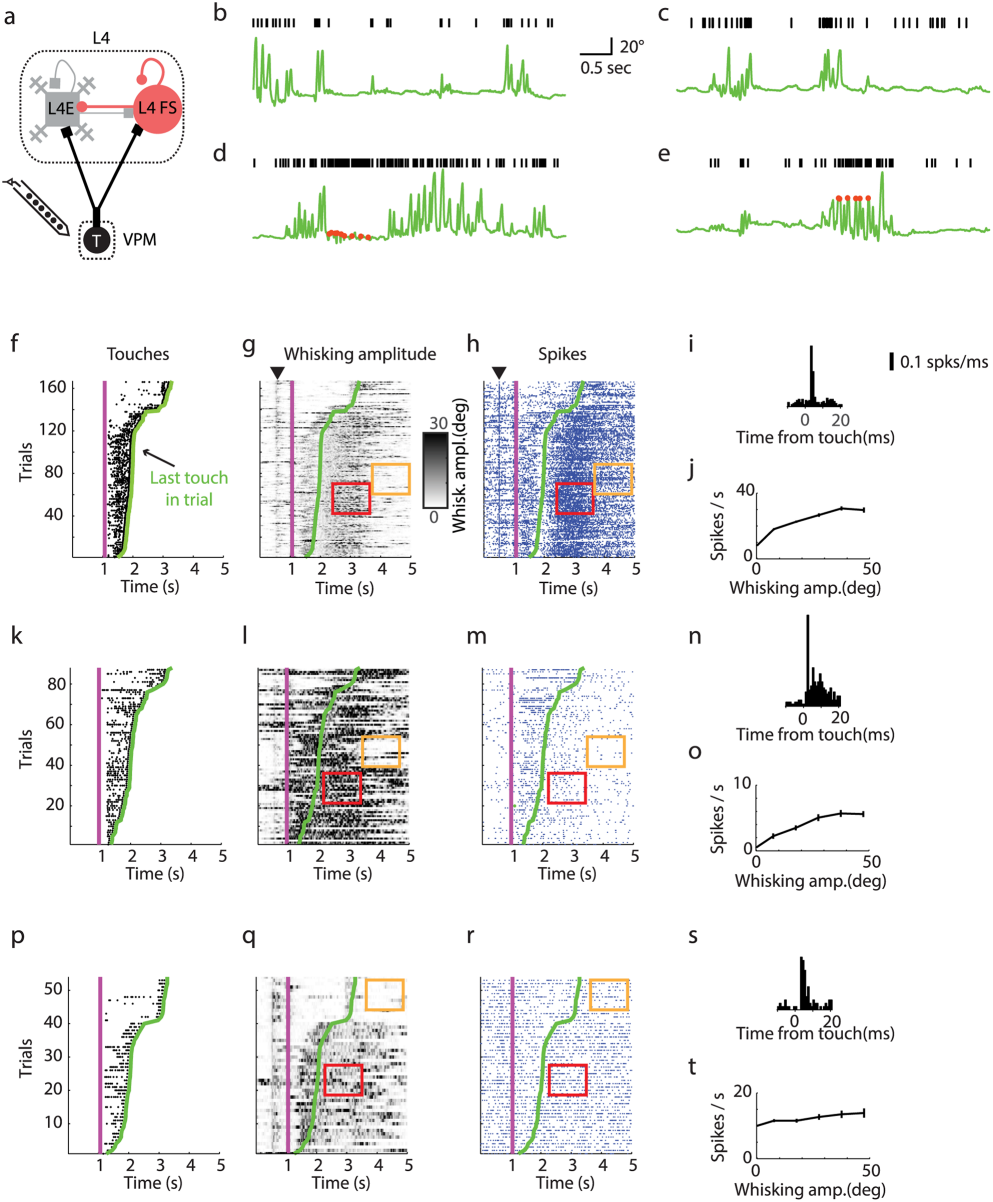
Example neurons recorded in VPM during whisker-based object localization. **(a)** Chronic silicon probe recordings in VPM. **(b**,**c)** Two example trials from the same neuron showing increases in activity with whisker movements without touch. Green line, whisker azimuthal angle. Black ticks, spikes. **(d**,**e)** Two example trials showing increases in spike rate after touch. **(f)** Touch rasters (black dots; sorted by last touch in a trial). The magenta line shows when the pole was moved within reach of the whiskers. The green line represents the last touch in each trial. **(g)** Whisker movement amplitude. Red rectangle, epoch of high whisker movement amplitude and high spike rate (same as in **h**). Orange rectangle, epoch of low amplitude and low spike rate (same as in **h**). **(h)** Spike raster for an example neuron in VPM (blue dots, spikes). The black arrow in **g-h** indicates onset of whisker twitching [52]. The twitching is triggered by an auditory signal generated by the brief activation of a shutter. **(i)** Peri-stimulus-time-histogram (PSTH) showing response for touch. **(j)** Spike rate as a function of whisker movement amplitude. **(k-o)** Same as **f-j** for another example VPM neuron with low baseline spike rate. **(p-t)** Same as **f-j** for another example VPM neuron that does not show modulation with whisking amplitude.

**Fig 3:**
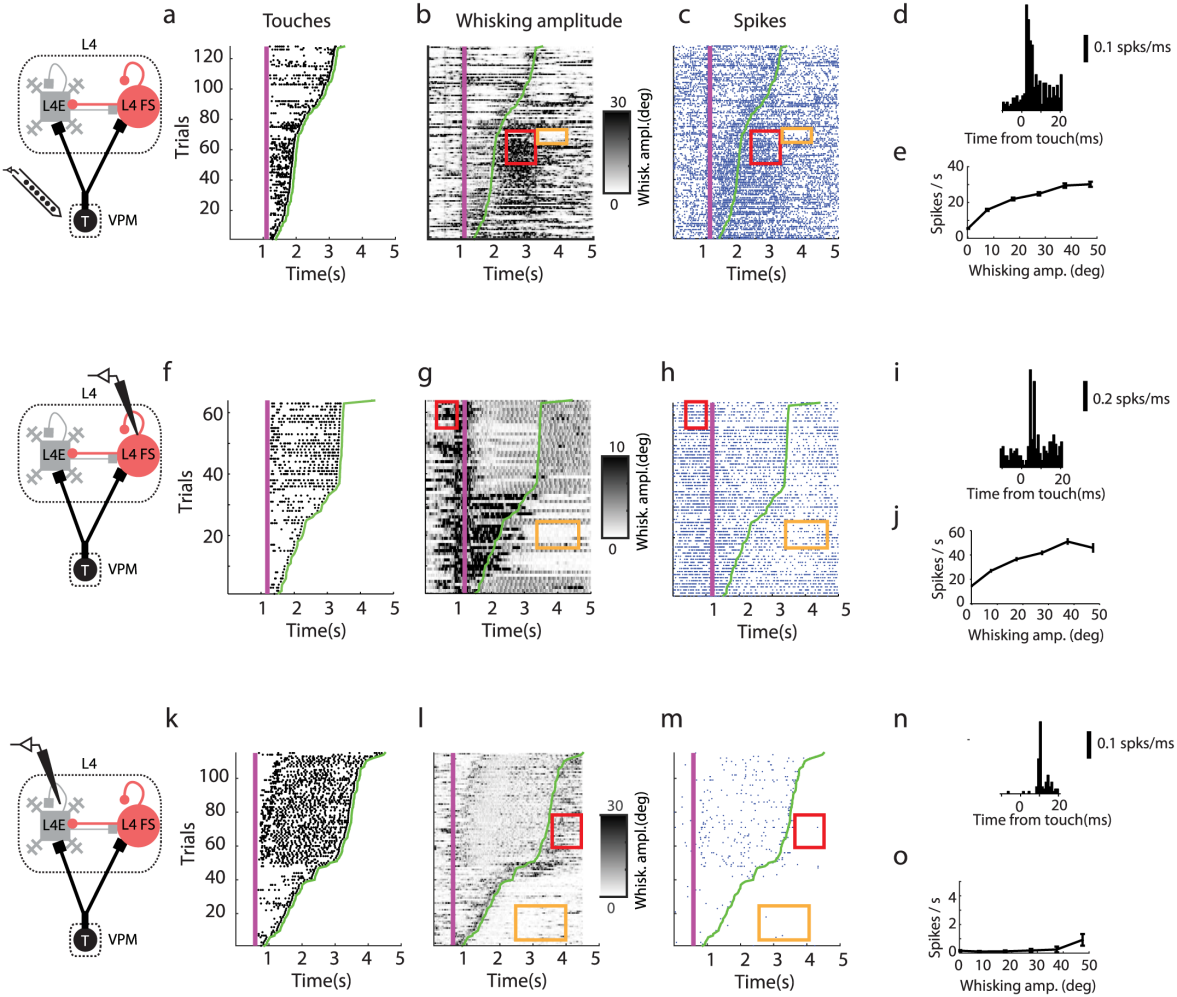
Example neurons across the thalamocortical circuit. **(a)** Touch raster (black dots; sorted by last touch in a trial). The magenta line shows when the pole was moved within reach of the whiskers. The green line represents the last touch in each trial. **(b)** Whisker movement amplitude. Red rectangle, epoch of high whisker movement amplitude and high spike rate. Orange rectangle, epoch of low amplitude and low spike rate. **(c)** Spike raster of an example neuron in VPM (blue dots, spikes). Red and orange regions of interest correspond to B. **(d)** PSTH showing response for touch. **(e)** Spike rate as a function of whisker movement amplitude. **(f-j)** Same as **a-e** for a L4 FS neuron. **(k-o)** Same as **a-f** for a L4 excitatory neuron. The few spikes that occur during whisking are phase-locked to movement.

VPM neurons have significant spike rates (mean, 5.1 Hz), even in the absence of whisker movement and touch (Table 1; Fig 1c and 1d; see Figs 2 and 3 for representative examples) [37]. VPM neurons were also highly sensitive to whisking (Fig 2b and 2c) [17, 18, 21]. Spike rates increased after whisking onset (average, 3-fold; Fig 1c and 1d). Given that mice whisk at approximately 15 Hz under these conditions [36], the spike rates during whisking correspond to approximately one spike per whisking cycle, on average. The modulation depths with whisking amplitude and phase (Supplementary Figure 2 in [37]) were similar to published studies in rats [18]. VPM neurons also responded to active touch with a brief increase in spike rate (0.6 spikes per touch) (Fig 1e). The peri-stimulus time histogram (PSTH) aligned to touch onset shows a sharp peak in spike rate, with short latency after touch (3.1±0.6 ms) and brief duration (2.9±1.7 ms) (Fig 2i, 2n and 2s). Exafferent touch signals and reafferent whisking signals were multiplexed in individual VPM neurons (Fig 2).

**Table 1:** Summary experimental results for VPM, L4 excitatory and L4 FS neurons. Data are taken from [37]. The increase of the average spike rate of L4 excitatory neurons from non-whisking to whisking conditions was found to be insignificant. Values are reported as mean ± standard deviation. In comparison to VPM neurons, the average response of L4 excitatory neurons is reduced by a factor of 2 in response to touch and by a factor of 20–30 in response to whisking onset.

Area	Figure	N	Non-whisking	Whisking	Spikes/touch
-	-	-	*Spks/s*	*spks/s*	*spks*
VPM	4	29	5±6	14±13	0.6±0.5
L4 FS	4	18	9±9	21±16	1.9±1.0
L4e	4	46	0.4±0.6	0.6±0.9	0.3±0.4

The modulation of L4 FS neurons to touch and whisker movement were similar to VPM neurons (Figs 1c–1e, 2 and 3). L4 FS neurons increased their spike rate after onset of whisking (average, 3-fold). L4 FS neurons responded reliably to touch. The response onset had slightly longer latency (5–15 ms) and longer duration (2–15 ms) than for VPM neurons. The increased latency is expected from the propagation time delay between VPM and L4 (2 ms) [37, 53].

The responses of L4 excitatory neurons differed profoundly from those of VPM and L4 FS neurons [36] (Figs 1c–1e and 3). The baseline spike rate of L4 excitatory neurons was much lower and did not increase significantly after onset of whisking. The response after touch onset occurred with longer latency and longer duration than for the VPM neurons, and similar to L4 fast-spiking neurons (Fig 1*e*).

The transformation performed by L4 circuits can be summarized by three main findings (Fig 1c–1e; Table 1). First, baseline spike rates are high in VPM and L4 FS neurons, and low in L4 excitatory neurons. Second, VPM and L4 FS neurons elevate their spike rates further during periods of whisker movement, whereas L4 excitatory neuron spike rates remain low. The spike rates of VPM and L4 FS neurons during periods of whisking are more than one order of magnitude larger than those of L4 excitatory neurons. Third, all three neuron types respond to touch reliably (Table 1; Fig 1e). The touch responses of the three neuronal populations are the integrals under the curve in Fig 1e. The average touch response of L4 excitatory neurons (0.3 spikes/touch) is about half of the average touch response of VPM neurons, whereas the spike rates of L4 excitatory neurons are lower than those of VPM neurons by a factor of 20–30. The VPM response is more transient than the L4E response. The L4 circuit therefore performs a behaviorally relevant computation by propagating information related to external stimuli and suppressing responses to whisker movement, a predictable stimulus.

**Fig 4:**
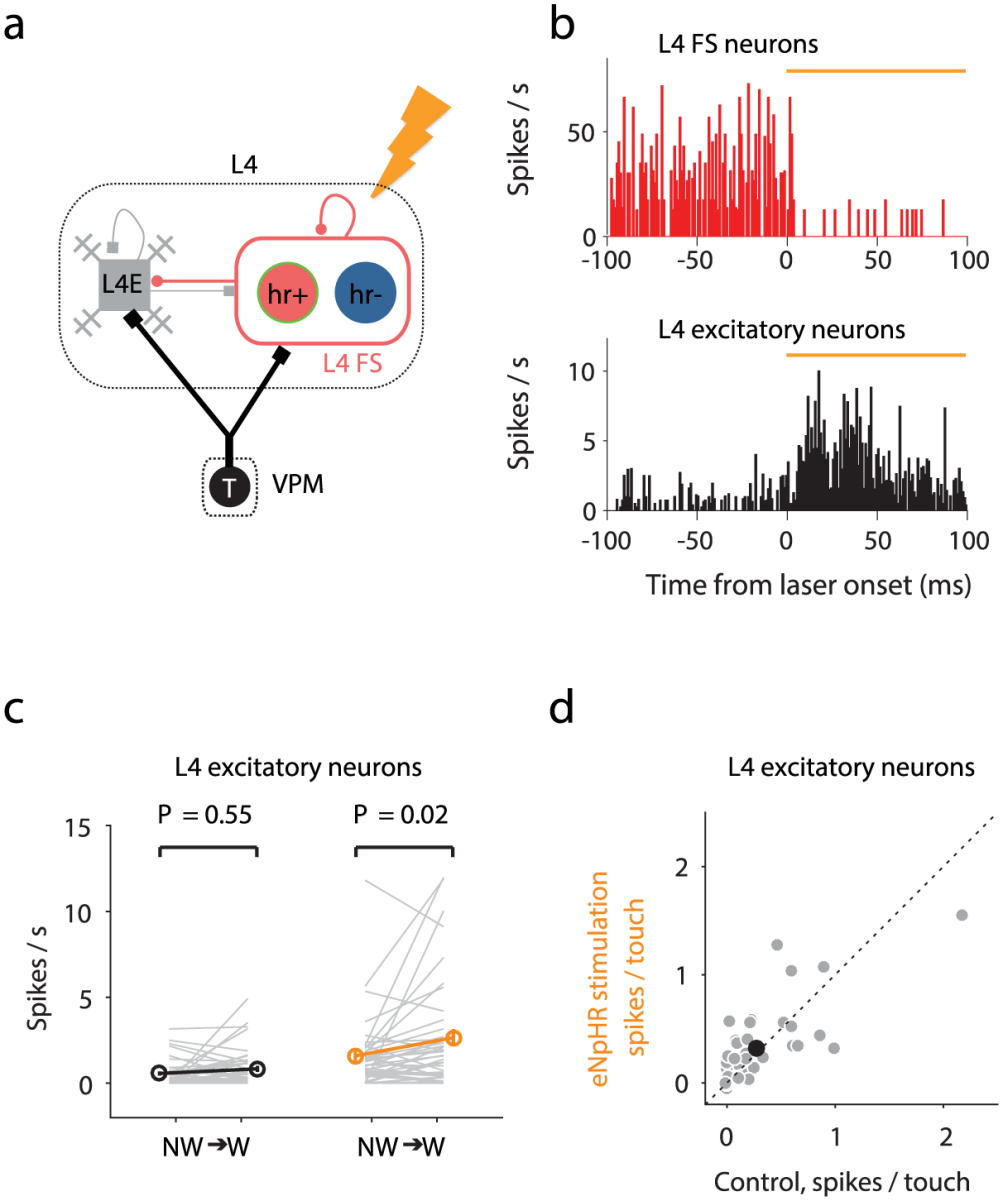
Summary of effect of photoinhibition of L4 FS neurons on L4 neurons. **(a)** Schematic of the Hr^+^ experiment. A subset of L4 FS neurons do not express Hr (Hr^-^ neurons). **(b)** Photostimulation of Hr^+^ neurons decreases the activity in L4 FS neurons and increases the activity in L4 excitatory neurons. Black: without photostimulation; orange: with photostimulation. **(c)** Response of L4 excitatory neurons during whisker movements (adapted from panel 8d in [37]). **(d)** Response to touch of L4 excitatory neurons. The black circle denotes the mean values (adapted from panel 8e in [37]).

The dynamics of FS neurons during whisker movement suggest that these neurons suppress whisker movement-related activity in L4 excitatory cells. Consistent with this view, optogenetic reduction of activity in a subset of L4 FS neurons expressing the light-gated, inhibitory chloride pump eNphHR3.0 (L4I-Hr^+^) unmasks movement-related activity in L4 excitatory neurons [37] (Fig 4*a* and 4*b*). Photostimulation of L4I-Hr^+^ neurons decreases the activity in L4 FS neurons on average and increases the activity in L4 excitatory neurons. The suppression of L4I-Hr^+^ neurons causes an increase in response in L4 excitatory neurons during whisker movements (Fig 4*c*) and an overall increase in the number of spikes per touch (Fig 4*d*). This result implies that suppression of whisking response in L4 excitatory neurons involves inhibition from FS neurons. It remains unclear, however, why touch responses in L4 excitatory neurons are not suppressed as well.

Membrane potential measurements with whole-cell recordings provide additional clues about mechanisms [37]. L4 excitatory neurons depolarize substantially (6 mV) and briefly after touch. Touch-related inhibitory input (from FS neurons) to L4 excitatory neurons is delayed by approximately 0.5 ms with respect to excitatory input (Fig 1e). It has been proposed that this short ‘window of opportunity’ [2, 35] allows L4 excitatory neurons to spike after touch, typically with one spike [36], before inhibition suppresses L4 excitatory activity. During whisker movement, excitation is matched by inhibitory input, keeping the L4 excitatory neuron membrane potential well below spike threshold. Suppression of self-movement signals is therefore implemented by inhibition within L4.

Yu et al. [37] also showed that activating halorhodopsin in L4I-Hr^+^ neurons during whisker movement suppresses activity in these neurons. This result poses a new question. The major cellular effect of activation of halorhodopsin is hyperpolarization [54, 55]. Theoretical investigations of cortical circuits, consisting of recurrently connected excitatory neurons stabilized by inhibitory neurons, have investigated the effects of hyperpolarization of inhibitory neurons [41]. Over a large range of conditions, hyperpolarization of inhibitory neurons leads to *increased* spike rates in both excitatory and also inhibitory neurons. This "paradoxical effect" is common to multiple network regimes, including networks with strong synaptic conductances that fire in an asynchronous manner [40] and networks with moderate synaptic conductances, if the excitatory-to-excitatory synaptic coupling *g*_EE_ is sufficiently strong [41]. Does the L4 circuit lie outside of the modeled parameter regimes, or can other factors explain the lack of paradoxical effect?

### A computational model of tactile suppression in L4

**Fig 5:**
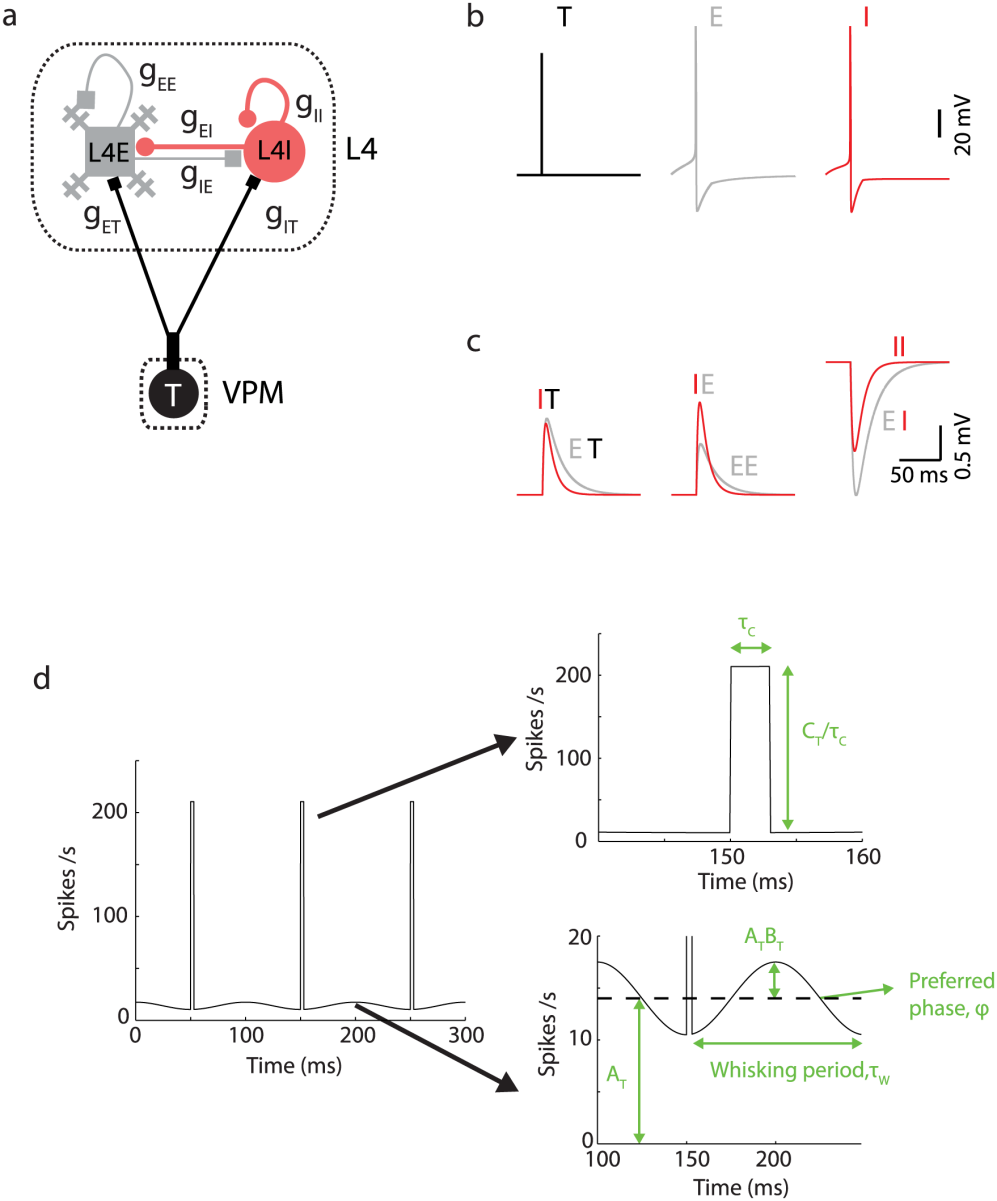
Neural network model of L4. **(a)** Diagram of the recurrent model of L4 network. **(b)** Spike shape of VPM (schematic), L4E and L4I neurons. **(c)** Temporal dynamics of individual EPSPs for the different synaptic connections (T = VPM; I = L4 FS; E = L4E). The convention is that that the first letter corresponds to the post-synaptic neuron and the second letter to the presynaptic neuron. **(d)** Thalamic generating function *F*_T_ (Eq 1). The panels on the right show the same figure in a magnified scale. For simplicity, we assume that all T neurons have the same preferred phase.

To explain the dynamical response of the L4 circuits to thalamic input, and to understand the roles of feedforward and recurrent connections, we constructed and analyzed a detailed computational model of the L4 circuit. L4 excitatory and L4 FS neurons make connections within type and across types with high connection probability (Fig 5*a*; Table 2). The thalamocortical circuit of the rodent whisker system is one of the most extensively studied mammalian circuits. *In vitro* and *in vivo* studies have measured many fundamental parameters that are required to construct a realistic computational model of the thalamocortical circuit. Our model circuit consists of 1600 L4 excitatory (E) neurons and 150 L4 FS (I) neurons [28] receiving input from 200 VPM (T) neurons (Fig 5a). When referring to modeling results we denote VPM neurons as T, L4 excitatory neurons as L4E, and GABAergic interneurons as L4I (Fig 5*b* and 5*c*). Since we know little about non-FS GABAergic interneurons during behavior we model only one inhibitory neuronal population.

**Table 2:** Network parameters in the reference parameter set: $\tau_{\text{delay}}^{\alpha \beta }$- synaptic delay, *K*_*αβ*_—average number of presynaptic inputs, *g*_*αβ*_—synaptic conductance, *V*_extr_—the extremal value of the unitary synaptic membrane potential change. The corresponding experimental values for *K*_*αβ*_ and *V*_extr_ are written in the two right columns. Those values are taken from the following references: a—[53], b—[56], c—[34], d- [28], e–[57], f—[27].

Populations	Synaptic Receptor	$\tau_{\text{delay}}^{\alpha \beta }$ (ms)	*K*_*αβ*_	*g*_*αβ*_ (mS/cm^2^)	*V*_extr_ (mV)	*K*_*αβ*_ experimental values	*V*_extr_ (mV) experimental values
ET	AMPA	1.0	50	0.15	1.1	100^a^	0.5^a^, 2.4±2^b^ (response to first spike in a train), ~1^b^ (spike train ~10 Hz).
IT	AMPA	1.0	75	0.2	1.03	150^c^	4.1±3^b^ (response to first spike in a train), ~1^b^ (spike train ~10 Hz), 3^c^
EE	AMPA	1.0	200	0.2	0.73	400^e^	1.1±1.1^b^, 0.52^d^, 1.6±1.6^e^
IE	AMPA	1.0	400	0.6	1.33	800^b^	2.2±2.2^b^
EI	GABA_A_	0.85	25	0.7	-1.92	50^b^	-1.1^b^, -1.0^f^
II	GABA_A_	0.5	25	0.55	-1.28	50^d^	-1.8^f^

Cortical neurons were simulated using a conductance-based model with a single compartment per neuron [58, 59]. Synaptic conductances are denoted as g_*αβ*_, where *β* is the presynaptic population and *α* is the postsynaptic population (Fig 5*a*). Model parameters, including numbers of neurons, unitary synaptic conductance, connection probability, resting potential, and membrane time constants are based on neurophysiological measurements in brain slices [27, 28, 56, 60] with small adjustments (see Methods). VPM provides the only external input to the model circuit, with connectivity estimates based on *in vivo* and brain slice measurements [34, 53]. Spike trains of T neurons were modeled as independent inhomogeneous Poisson processes with a generating function *F*_T_ representing thalamic activity during quiescence, whisking or whisking and touch (Fig 5*d*; see Methods).

$$F_{\text{T}}(t)=A_{\text{T}}\left[1+B_{\text{T}}\text{sin}\left(\frac{2\pi t}{\tau_{w}}+\phi \right)\right]+\frac{C_{\text{T}}}{\tau_{c}}\Theta (t-n\tau_{w}-t_{c})\Theta (n\tau_{w}+t_{c}+\tau_{c}-t)$$ (1)

where *A*_T_ is the spike rate averaged over a whisker movement cycle, *B*_T_ is the modulation depth, and *C*_T_ is the number of spikes per touch, *τ*_*w*_ is the whisking period, *t*_c_ is the time of touch onset within a whisking cycle, *n* is the cycle number, and Θ is the Heaviside function. During whisker movements *A*_T_ increases above a baseline, with sinusoidal modulation phase-locked to a single preferred phase *ϕ*. Touch is represented by adding a rectangular function at touch onset *t* = *t*_c_ (with respect to the whisking cycle), stretched over *τ*_*c*_ = 3 ms with an integral of *C*_T_ = 0.6 spikes per touch (Eq 1). Correlations between thalamic neurons beyond those generated by Eq 1 have not been measured and were thus not modeled. The population-average spike rate of neurons within a population over whisking cycles is denoted by ν_*α*_ (*α* = T,E,I), and the population-average spiking response to touch is denoted by *R*_*α*_. Note that without touch, ν_T_ = A_T_. A ‘reference parameter set’ is described in Methods and used unless otherwise stated.

### Response of the model circuit to whisker movement and touch

**Fig 6:**
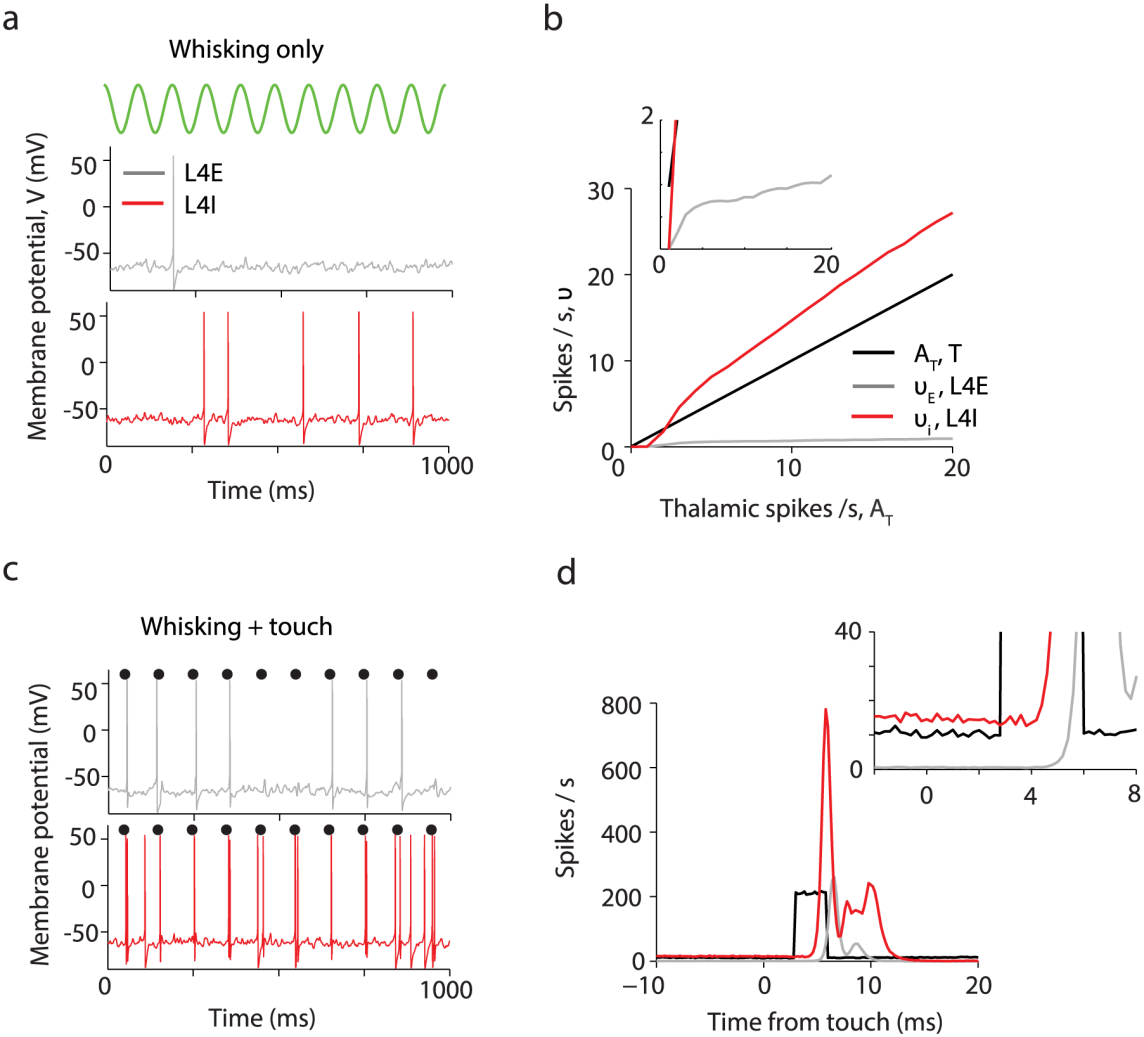
A neural network model of L4 explains suppression of whisker movement signals in L4 excitatory neurons. The colors black, grey and red denote T, L4E and L4I neuronal populations respectively. **(a)** Example L4E and L4I membrane potential during simulated whisking (green). **(b)** The population- and time average spike rates ν_E_ and ν_I_ of the L4E and L4I neurons respectively as function of the thalamic input *A*_T_ in the absence of touch. L4I neurons follow linearly the thalamic input while L4E neurons increase only weakly with *A*_T_ beyond firing threshold. Inset, zoom in. **(c)** Membrane potential for an example neuron during whisking and touch (black dots). **(d)** Population PSTH aligned to touch onset. Inset, zoom in.

We start by simulating the model for whisking without touch (thalamic spikes per touch, C_T_ = 0). L4E spike rates were low (ν_E_ < 1 Hz) compared to those of L4I neurons (Fig 6*a* and 6*b*). The average rates ν_E_ and ν_I_ depend on *A*_*T*_ (the population- and time-average thalamic spike rate during whisking only) but are almost independent of the modulation depth B_*T*_ (Eq 1). Except near threshold, *ν*_I_ was proportional to *A*_*T*_, in consistent with theoretical results for large and sparse neuronal networks with strong synapses, in which strong excitation is compensated by inhibition (balanced networks) [38]. The L4E spike rate *ν*_E_ increased linearly with *A*_*T*_, but with a much smaller slope compared to *ν*_I_.

The linearity of the *ν*_I_-*A*_*T*_ curve fit empirical observations (Table 1, Fig 1*c*; spike rates of both VPM and L4 FS neurons more than double in the transition from non-whisking to whisking). The average spike rate of L4 excitatory neurons, *ν*_E_, is low [37]. During transition from non-whisking to whisking, the average spike rate of L4 excitatory neurons barely changes (from 0.4 Hz to 0.6 Hz; Table 1). The spike rates of L4E neurons in our model show similar dynamics.

**Fig 7:**
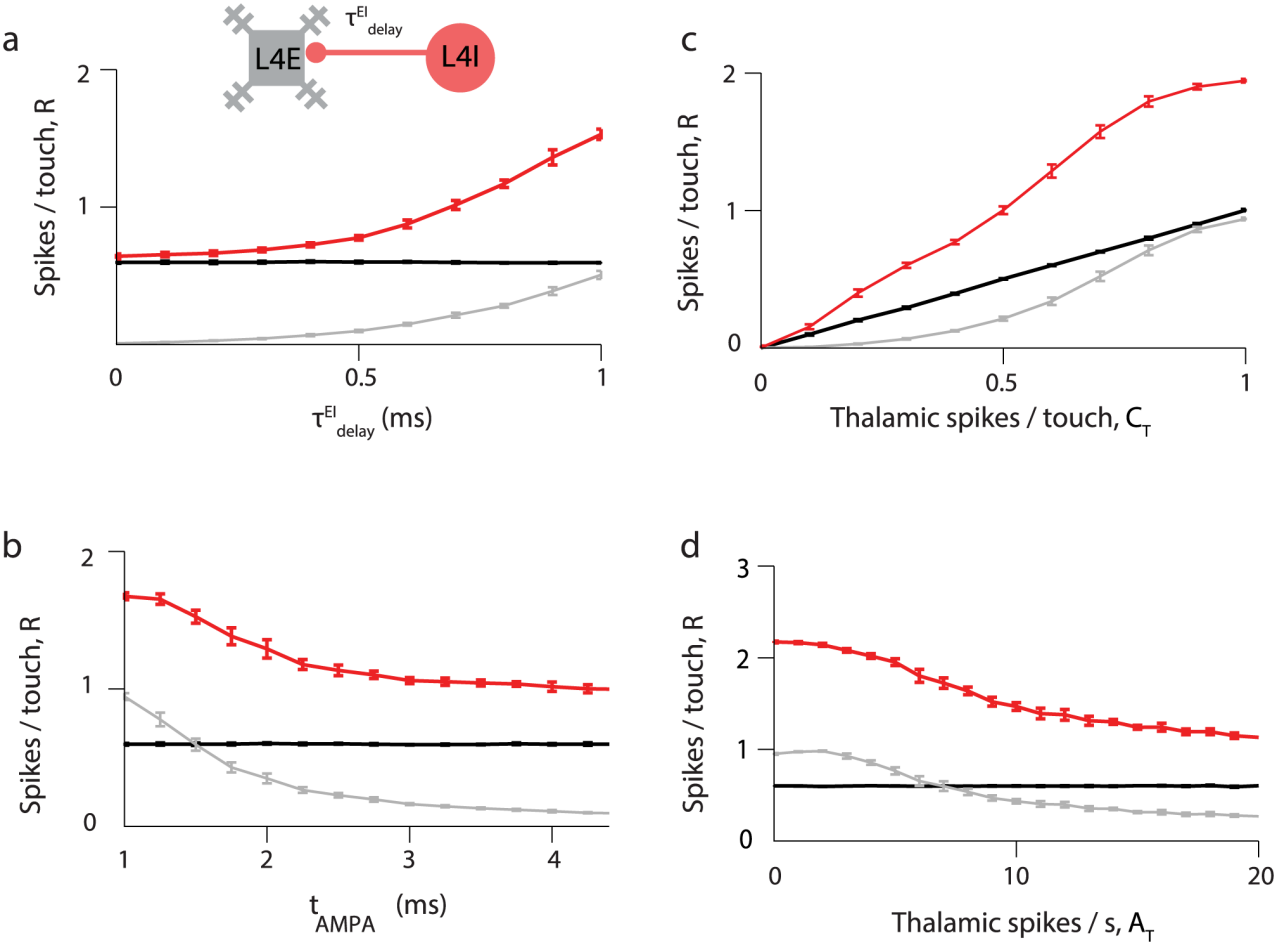
Touch response in function of synaptic delay, AMPA receptors’ time constant and parameters defining thalamic input. In each panel, responses to touch in L4E and L4I neurons (*R*_E_ and *R*_I_, in grey and red respectively) are plotted, as well as the thalamic response in black. Spikes per touch were counted up to 25 ms after touch onset, and baseline computed by counting spikes 25 ms before touch is subtracted. Responses to touch are plotted as functions of **(a)** I-to-E synaptic delay $\tau_{\text{delay}}^{\text{EI}}$, **(b)** the AMPA receptor time constant *t*_AMPA_, **(c)** the thalamic response to touch, *C*_T_, and **(d)** the thalamic spike rate *A*_T_ during whisker movements without touch.

**Fig 8:**
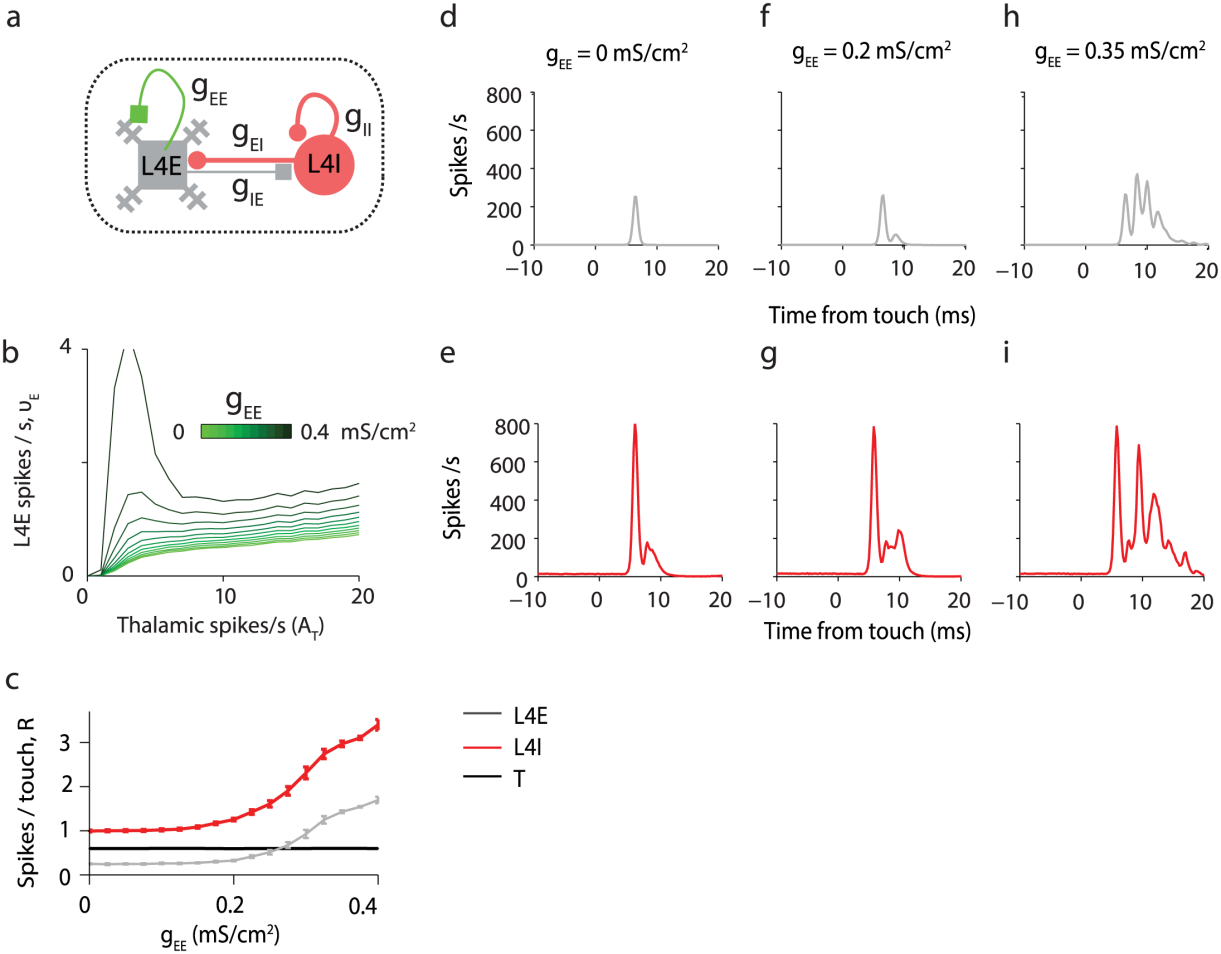
Effects of varying intracortical recurrent excitatory conductances *g*_EE_ on the function of L4E neurons. **(a)** The circuit with changing g_EE_ emphasized in green. **(b)** ν_E_ vs. *A*_T_ during whisker movements only, for 11 values of *g*_EE_ from 0 (light green) to 0.4 mS/cm^2^, that is twice the reference parameter value (dark green). Recurrent excitation *g*_EE_ increases ν_E_ while not affecting the slope of the ν_E_-*A*_T_ curve far from spiking threshold substantially. **(c)** *R*_T_, *R*_E_ and *R*_I_ as functions of *g*_EE_. Other parameters: *A*_T_ = 14 spikes/s, *C*_T_ = 0.6. **(d)** PSTH aligned to touch onset for L4E and without recurrent excitation (*g*_EE_ = 0 mS/cm^2^). **(e)** Same as **C** for L4I. **(f-g)** Same as **c**-**d** for *g*_EE_ = 0.2 mS/cm^2^. **(h-i)**: Same as **c**-**d** for *g*_EE_ = 0.35 mS/cm^2^. Beyond *~g*_EE_ = 0.4 mS/cm^2^ the network exhibits runaway excitation.

L4E neurons fire at most one spike each after touch, whereas L4I neurons fire more than one (up to two) spikes per touch (Fig 6*c* and 6*d*). The synaptic delay, $\tau_{\text{delay}}^{\text{EI}}$, is a critical factor in determining the strength of the touch response (Fig 7a). For $\tau_{\text{delay}}^{\text{EI}}=0$, the average normalized response of L4E neurons after touch, *R*_E_, is small (*R*_E_ = 0.01 spikes/touch), and L4I neurons fire *R*_I_ = 0.64 spikes/touch. Strong inhibition overwhelms excitation before L4E neurons have a chance to spike. The delay between the appearance of excitation and feed-forward inhibition in the L4E neuron has been termed ‘window of opportunity’ [2, 35, 61–63]. This ‘window of opportunity’ increases with $\tau_{\text{delay}}^{\text{EI}}$, but is not equal to it because it is affected by the durations of thalamocortical synaptic process and the time needed for the L4I to fire in response to the brief and strong thalamic input. For a more realistic value, $\tau_{\text{delay}}^{\text{EI}}=0.85\text{ms}$, L4E and L4I fire 0.34±0.24 and 1.3±0.07 spikes/touch respectively, similar to experimental measurements (Table 1). The touch responses for L4E and L4I (Fig 6d) are narrower than those seen in real data (Fig 1e). The detailed shapes of the modeled responses depend on network parameters (*e*.*g*., see Fig 8d–8i below). In addition, heterogeneity among neurons, which was not modeled here, is expected to broaden the responses.

The response of the network to touch depends on the time-course of synaptic conductances. Without touch signals, L4E neurons are inhibited by L4I neurons and spike at low rates (Fig 6*a* and 6*b*). Touch produces strong, brief and synchronous thalamic excitation (Fig 1*e*), which depolarizes L4E neurons and enables them to fire before inhibition terminates the response. This mechanism demands that excitatory synaptic conductance changes at thalamocortical synapses are brief. Touch responses of L4E and L4I neurons decrease with *t*_AMPA_, the decay time of AMPA-mediated EPSCs (Fig 7*b*). Substantial touch responses in L4E neurons require *t*_AMPA_ < 2–3 ms, consistent with the brief excitatory conductances measured at thalamocortical synapses [60].

Touch responses of L4E and L4I neurons increased with the strength of the thalamic touch signal (Fig 7*c*), consistent with graded responses to touch strength measured in L4E neurons [36]. The response saturates at one and two spikes/touch for L4E and L4I respectively; this is in part due to the intrinsic properties of these neurons, which preclude them from firing more spikes in response to brief thalamic input.

We have shown that a L4 network with parameters similar to experimentally determined values can replicate major experimental findings. L4E excitatory neurons exhibit low baseline activity, low activity in response whisker movement, and significant response to touch. However, during behavior the responses of L4 excitatory neurons is not all-or-none: L4 excitatory neurons are tuned to multiple sensory features including touch direction, intercontact interval, strength of touch, and likely other factors [36]. Our simulation results indicate that L4E and L4I neurons respond to touch in a graded manner and robustly respond to touch at different whisking amplitudes. Specifically, our simulations show that *R*_E_ and *R*_I_ decrease with *A*_T_ (Fig 7*d*) for all parameter values consistent with the data. Mechanistically, increasing thalamic input causes larger inhibition in the cortical circuit that decreases touch response. Future experiments could test this model prediction.

### Contributions of intracortical connections in tactile suppression

The model allows us to explore how specific synaptic connections within L4 contribute to fine-tuning L4 function. These connections currently cannot be specifically manipulated, making them difficult to evaluate experimentally. Recurrent excitation (*g*_EE_) can only be tuned over a limited range before runaway excitation is triggered (for about *g*_EE_~0.4 mS/cm^2^), even during baseline or whisking (Fig 8*a* and 8*b*). The conductance *g*_EE_ does not modify the slope of the ν_E_-*A*_T_ curve (Fig 8*b*), but shifts this curve upward. *g*_EE_ amplifies touch responses *R*_E_ and *R*_I_ (Fig 8*c*) [64], mostly by increasing the duration of touch responses (Fig 8*d*–8*i*).

**Fig 9:**
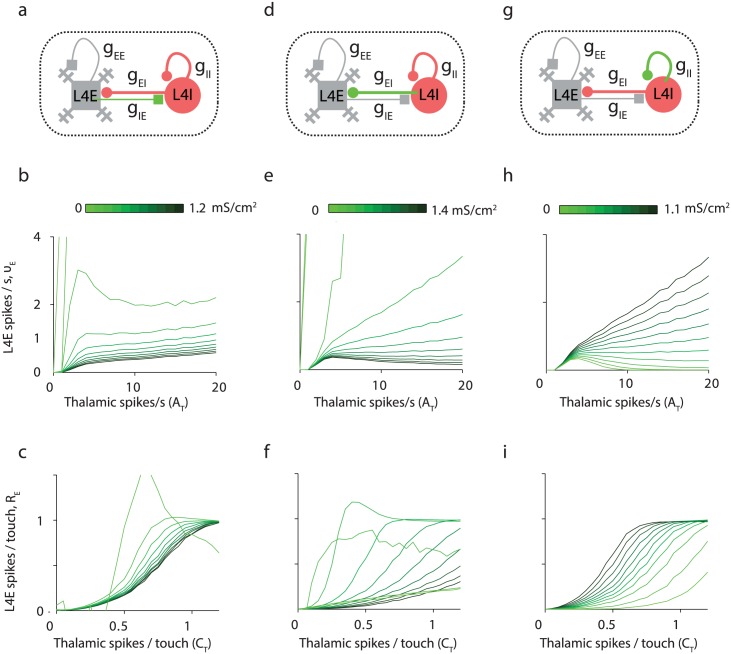
Inhibition in L4 controls the response to L4E neurons. **(a-c)** Changing *g*_IE_ from 0 to 1.2 mS/cm^2^ **(d-f)** Changing *g*_EI_ from 0 to 1.4 mS/cm^2^. **(g-i)** Changing *g*_II_ from 0 to 1.1 mS/cm^2^. In the top panels, the synaptic connection that its strength is varied is plotted in green. In the middle and bottom panels, curves are plotted for 11 values of the relevant *g* from 0 (light green) to its maximal value, that is twice the reference parameter value (dark green). **(b**, **e**, **h)** The response of L4E neurons, ν_E_, to slowly varying thalamic input, which correlates with the amplitude of whisking. **(c**, **f**, **i)** The response of L4E neurons to spikes, R_E_, associated with touch.

The dynamics of L4E neurons are shaped in subtle ways by L4 inhibition. Fig 9 shows how parameters involving inhibition (*g*_II_, *g*_EI_, *g*_IE_) affect circuit responses, while holding the other parameters at their reference values. Intracortical excitation of inhibition (*g*_IE_) is necessary to prevent runaway excitation, and thus keeps the spike rates of neurons in the network moderate (Fig 9a and 9b). Reducing *g*_IE_ shifts the ν_E_-*A*_T_ curve during whisking upward, without modifying its slope. For touch responses, *g*_IE_ shifts the touch-response *R*_E_-*C*_T_ curve to the right while decreasing the slope of the linear section of this sigmoid (Fig 9*c*).

**Fig 10:**
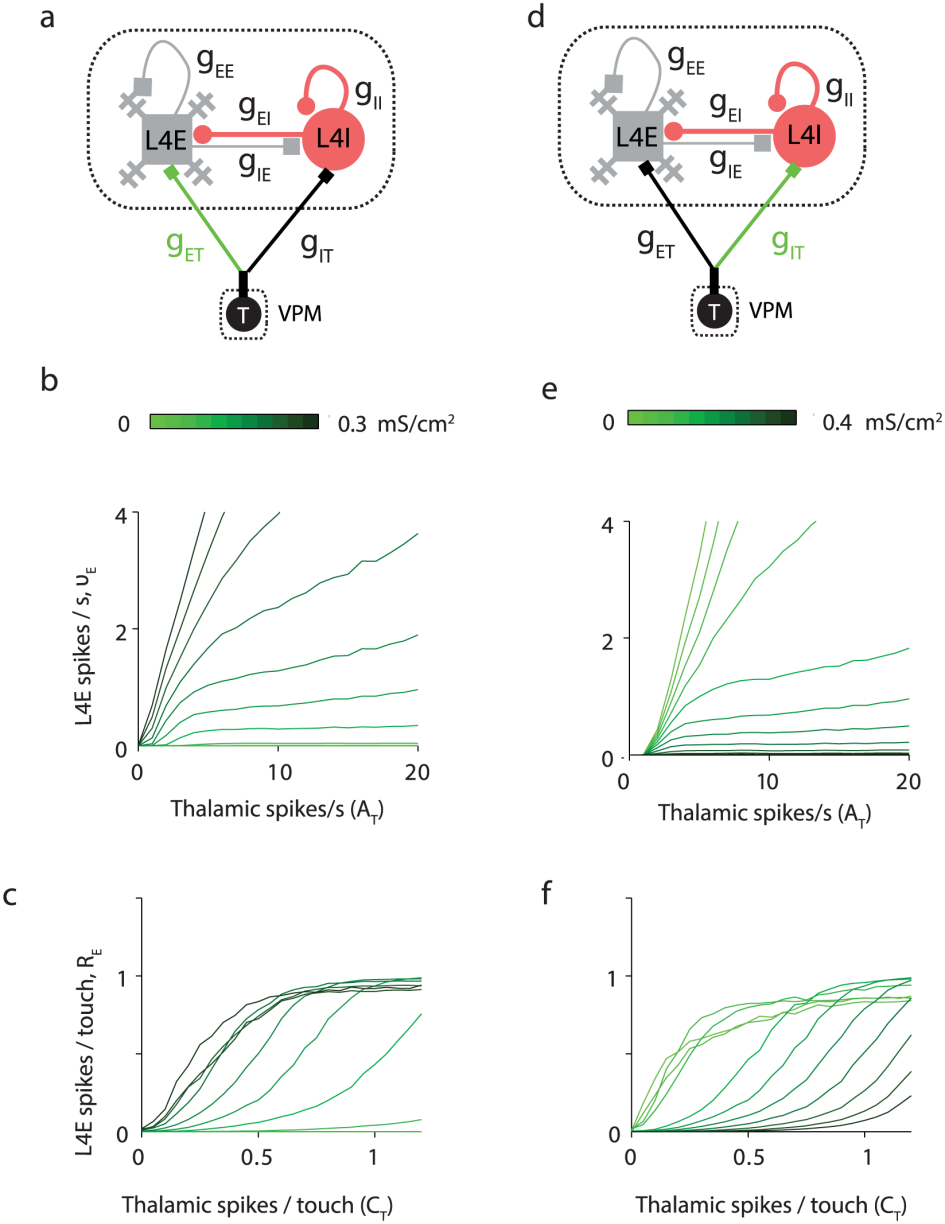
Effect of varying thalamocortical conductances on the function of L4E neurons. Symblols and lines are as in Fig 9. **(a)** Changing *g*_ET_. **(b)** ν_E_ vs. *A*_T_ during whisker movements only. **(c)** R_E_ vs. *C*_T_. **(d)** Changing *g*_IT_. **(e-f)** Same as **b-c**.

Inhibition to L4E neurons (*g*_EI_) is also required to prevent runaway excitation (Fig 9*d* and 9*e*). For low values of *g*_EI_ (but above values for runaway excitation) and large values of *g*_II_ (Fig 9*g* and 9*h*), *ν*_E_ scales linearly with thalamic input *A*_T_. This linear scaling is known from balanced networks with strong synaptic coupling [38, 40]. The situation is different for sufficiently strong inhibition (relatively high *g*_EI_, moderate *g*_II_), where the response of L4E neurons to slowly-varying thalamic input during whisking, *ν*_E_, is independent, and even decreasing, with *A*_T_ (Fig 9*e* and 9*h*). For small enough *g*_II_, L4E are quiescent except of near firing threshold. In addition, low *g*_ET_ and large *g*_IT_ values are critical to keep *ν*_E_ low for all whisker movement amplitudes (Fig 10*a*, 10*b*, 10*d* and 10*e*). Furthermore, these low values *ν*_E_ are nearly independent of *A*_T_. This regime is consistent with experimental data, where L4 FS neurons increase their spike rate with whisker movement, whereas L4 excitatory neuron spike rates remain low (Fig 1*d*, Table 1).

Increasing values of *g*_EI_ and *g*_II_ shifts the touch-response *R*_E_-*C*_T_ curve to the right and to the left respectively (Fig 9*f* and 9*i*). In addition, *g*_EI_, but not *g*_II_, decreases the slope of the linear section of this sigmoid (Fig 9*f*). Similarly, *g*_ET_ and *g*_IT_ shift the *R*_E_*-C*_T_ curve to the left and to the right respectively (Fig 10*c* and 10*f*).

### The effects of halorhodopsin activation in L4 FS neurons

Yu et al. ([37], Fig 8, supplementary Fig. 13) expressed halorhodopsin in FS neurons in L4 and explored how the responses of excitatory and FS neurons in L4 under baseline conditions, whisking and touch vary under halorhodopsin activation. L4 excitatory neurons increase their spike rates and L4 FS neurons decrease their spike rates, both during baseline and whisking conditions. Similarly, L4 excitatory neurons increase their spiking responses to touch and L4 FS neurons decrease their responses.

The halorhodopsin expression levels in FS neurons likely varied widely across individual neurons [65]. We therefore divided the L4I neurons in the model into hr expressing neurons and non-expressing neurons (Hr^+^ and Hr^-^ respectively). The fraction of Hr^+^ neurons among L4I neurons is denoted by *f*_halo_.

Manipulation of the components of neural networks can cause complex and counterintuitive change in network dynamics (‘paradoxical’ response; [40–42, 66]: injecting negative current to all inhibitory neurons in a network, that includes spiking excitatory neurons, increases the average spike rates in inhibitory neurons). If the synaptic conductance strengths are moderate and the excitatory-to-excitatory synaptic conductance is above a certain level, injecting negative current to the inhibitory neurons causes the spike rates of excitatory neurons *ν*_E_ to increase, and as a result the network dynamics causes the spike rates of inhibitory neurons *ν*_I_ to increase as well [41, 42, 66]. Alternatively, if synaptic conductances are strong and the excitatory population is active, the condition that the activity of excitatory neurons should be moderate (non-zero and not epileptic) causes *ν*_I_ to increase under negative current injection to inhibitory interneurons [40]. The major effect of halorodopsin is to hyperpolarize FS neurons (see Methods). Therefore, if all inhibitory neurons in the model are assumed to express halorodopsin equally (*f*_halo_ = 1), halorhodopsin activation will cause both L4E and L4I neurons to *increase* their spike rates.

**Fig 11:**
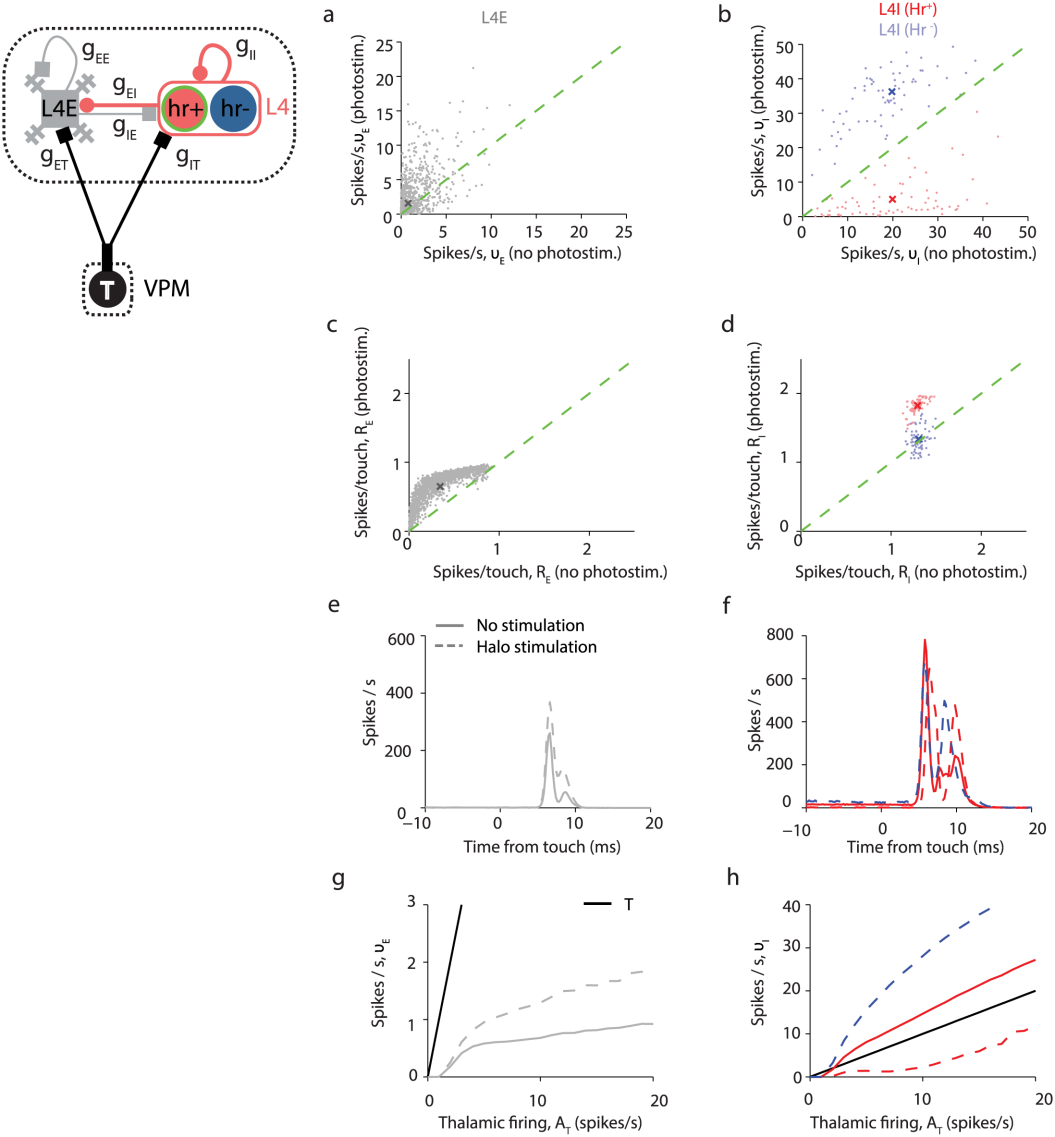
Simulated light activation of halorhodopsin expressed in L4I-Hr^+^ neurons. Simulations with *f*_halo_ = 0.5 reveals a reduction in the whisking suppression and an enhancement of touch responses by L4E neurons. **(a)** Halorhodopsin activation in L4I-Hr^+^ causes an average increase in response of L4E during whisking and no touch, with a wide distribution of halorhodopsin—induced modifications. **(b)** Most L4I-Hr^+^ neurons reduce their activity during whisking while L4I-Hr^-^ neurons increase it. **(c)** Increase in the touch responses in L4E neurons during suppression of L4I-Hr^+^. **(d)** Increase in the touch responses in L4I neurons. The increase in touch responses is only seen in Hr^+^ cells. **(e**,**f)** Population PSTH of L4E (**e**) and L4I (**f**) neurons with and without L4I-Hr^+^ activity suppression. **(g)** Reduction of L4I-Hr^+^ activity diminishes the whisking suppression effect in L4E neurons. Black line: T neurons; solid grey line: L4E neurons without halorhodopsin activation; dashed grey line: L4E neurons during halorhodopsin activation. **(h)** Reduction of L4I-Hr^+^ activity diminishes the whisking response in L4I-Hr^+^ neurons. Solid red line: L4I-Hr^+^ neurons without halorhodopsin activation; dashed red line: L4I-Hr^+^ neurons during halorhodopsin activation. Dashed blue line: L4I-Hr^-^ neurons during halorhodopsin activation.

We simulated the response of L4E and L4I neurons to whisking for *f*_halo_ = 1 without and with halorhodopsin activation, and replicated the 'paradoxical effect': the average responses of L4E and L4I neurons to whisking, *ν*_E_ and *ν*_I_, increase with simulated light activation. The simulation result for L4I neurons is in contrast to experimental observations showing that most L4 FS neurons increase their spike rates. This discrepancy is resolved if we assume *f*_halo_ = 0.5. For this value, spike rates during whisking increase, on average, for L4E neurons (Fig 11*a*), decrease for almost all L4I-Hr^+^ neurons (Fig 11*b*), and increase for inhibitory neurons that do not express halorhodopsin (L4I-Hr^-^).

The spike responses of L4E neurons to touch increase moderately for *f*_halo_ = 0.5, especially for neurons with low baseline response (Fig 11*c* and 11*e*), whereas the spike response of L4I-Hr^-^ inhibitory neurons remains about the same and that of L4I-Hr^+^ increases somewhat (Fig 11*d* and 11*f*). These dynamics stem from the fact that the initial response to touch, before feedforward inhibition hyperpolarizes the neuron, is determined mainly by feedforward excitation. While halorhodopsin activation hyperpolarizes inhibitory neurons and increase the difference between spiking threshold and their membrane potentials before touch onset, the driving force of excitation (the difference between the reversal potential of APMA-mediated excitation and their membrane potential) increases as well and enhances excitation.

The reduction in response in L4I-Hr^+^ neurons partially disrupts whisker movement suppression in L4E neurons, and increases the slope of the ν_E_-*A*_T_ curve (Fig 11*g*). During photostimulation of L4I-Hr^+^ neurons, their response to increasing whisker movements becomes shallower (Fig 11*h*). This change in response of L4-Hr^+^ neurons in turn causes a steeper response to whisker movements in L4E neurons (Fig 11*g*).

**Fig 12:**
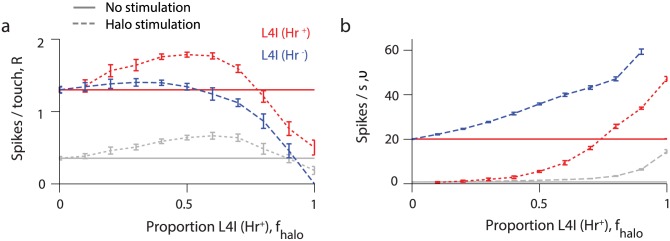
Effects of light activation of halorhodopsin expressed in L4I-Hr^+^ neurons. Effects of halorhodopsin light activation on touch response and whisking response for the L4E (grey), L4I-Hr^+^ (red) and L4I-Hr^-^ (blue) neuronal populations are plotted as functions of *f*_halo_, the fraction of L4I-Hr^+^ neurons among all L4I neurons. In both panels, values without and with light activation are denoted by solid and dashed lines, respectively. **(a)** Spikes per touch, *R*, for L4 neuronal populations as functions of *f*_halo_. **(b)** Average spike rates, ν, during whisking for L4 neurons as functions of *f*_halo_.

Finally we explored how the previous results depend on *f*_halo_. The mean touch responses for all neuronal populations is non-monotonic: it increases when *f*_halo_ increases from zero, and then decreases for higher values (Fig 12*a*). For our parameter set, simulated halorhodopsin activation increases touch response of L4I-Hr^+^ neurons (in comparison with no activation) for *f*_halo_ < 0.78. During whisker movements, the spike rates of all neuronal populations increases with *f*_halo_, and the average spike rate of L4I-Hr^+^ neurons during simulated halorhodopsin activation is lower than that with no activation for *f*_halo_ < 0.74 (Fig 12*b*). Interestingly, L4-Hr^+^ neurons reduce their spiking response to touch with halorhodopsin activation for large *f*_halo_, but reduce their spike rates in response to whisking for small *f*_halo_. For small *f*_halo_, the spike rates of L4-Hr^+^ neurons in response to whisking are low (Fig 12b), because this small group of neurons is both suppressed by halorhodopsin activation and inhibited by the majority of L4-Hr^-^ neurons. In contrast, the spiking responses to touch of L4-Hr^+^ and L4-Hr^-^ neurons are similar and to the responses of L4I neurons without halorhodopsin activation. This behavior is obtained because touch responses are transient and are reduced by the global level of inhibition before touch. For low *f*_halo_, population inhibition is similar to inhibition without halorhodopsin activation.

## Discussion

We computationally dissected how information is transformed between thalamus and barrel cortex at the level of defined neural circuits [36, 37] (Fig 1). Our model defines the mechanisms that enable the L4 circuit to transmit touch-related information and suppress self-motion signals in L4. Here we summarize our main modeling results and how they explain major experimental observations.

### Transmission of touch signals while suppressing whisking signals

We compared our model to recordings from VPM projection neurons (Figs 1–3), L4 FS neurons, and L4 excitatory neurons [37] during performance of an object localization task, while whisker movements and touches were tracked with millisecond time scale precision [36, 48, 51, 67]. VPM and L4 FS neurons respond to whisker movement and touch, whereas L4 excitatory neurons responded almost exclusively to touch. During whisking, excitation to L4 excitatory neurons from VPM is only slowly modulated in time and is matched by feedforward and feedback inhibition from L4 FS neurons, which cancels self-movement signals. In contrast to the self-movement input, touch-related inputs are brief and synchronous (Fig 1e). Our model establishes the conditions for a ‘window of opportunity’, in which L4 excitatory neurons can fire before inhibition catches up [35, 62, 64, 68, 69]. The I-to-E synaptic delay $\tau_{\text{delay}}^{\text{EI}}$ must be sufficiently large (say, ~1 ms) and the AMPA-mediated synaptic conductances should be brief (Fig 8a and 8b). The brief duration of this window diminishes the chance of L4 excitatory neurons to fire multiple spikes upon touch, producing low trial-to-trial variability in spike count after touch [36, 70].

### Spike rates during baseline and whisking

During baseline and whisking, L4 FS neurons spike on average at tens of Hz, while L4 excitatory neurons spike on average below 1Hz. The average spike rates of VPM and L4 FS excitatory neurons, *ν*_T_ and *ν*_I_, more than double after whisking onset, while the average spike rates of L4 excitatory neurons remains below 1 Hz. Low spike rates of L4E neurons require particular combinations of synaptic parameters: g_IE_, g_EI_ and g_IT_ need to be strong, and g_EE_, g_II_ and g_ET_ should be weak (Figs 8*c*, 9, 10). If g_ET_ is too small or g_IT_ is too large, L4E neurons will be quiescent. Similarly, L4E neurons will not spike if g_II_ is too small (Fig 9h). For moderate g_II_, *ν*_E_ can remain about constant with *A*_T_.

### Touch responses

The response of L4E neurons to touch, *R*_E_, increases with *C*_T_ (the thalamic response to touch) in a sigmoid manner, because the intrinsic properties of L4E neurons in the model impose a refractory period that in general precludes rapid firing of more than one spike. Synaptic parameters that increase *ν*_E_ during whisking shift the sigmoid function leftward, and those that decrease *ν*_E_ shift that function rightward (Figs 9 and 10). In our model, the inhibitory neuronal population spikes at significant rates (20–40 Hz) even at baseline. This allows the number of spikes/touch *R*_E_ to be smaller than one and vary gradually with input strength (Fig 9*f*). As a result, stronger thalamic responses to touch, for example as a result of stronger touch, generates proportionally stronger L4E responses, consistent with experimental results (Fig 1e) [36].

### Effects of photostimulation of L4-Hr^+^ neurons

In response to halorhodopsin activation, L4 excitatory neurons increase their average spike rates and L4-Hr^+^ FS neurons reduce their spike rates [37]. The model does not replicate this behavior if halorhodopsin is "expressed" in all inhibitory neurons, because *ν*_I_ increases with simulated photostimulation. This discrepancy is resolved if a sufficient number of L4I neurons lack halorhodopsin (or express halorhodopsin at very low levels) (Figs 11 and 12*b*). With photostimulation, L4-Hr^+^ I neurons decrease their spike rates if their fraction among I neurons, *f*_halo_, is below a certain value (0.74 in Fig 12*a*), while L4-Hr^-^ FS neurons increase their spike rates. Future work is needed to determine whether including more populations of inhibitory interneurons, for example somatostatin-positive neurons [71] will generate parameter regimes in which L4-Hr^+^ neurons decrease their responses to both whisking and touch upon light activation.

### Modeling approach

The development of powerful computing resources has enabled simulations of entire cortical columns of diverse neurons with realistic morphologies and biophysically plausible membrane conductances [72, 73]. Because of the profusion of parameters, detailed models are difficult to analyze to extract the underlying principles. So far these detailed models have failed to explain any neural computation [74]. Here we took a different approach. We implemented a computational model based on hard won numbers for connection probability, connection strength, neuron numbers and basic cellular parameters for the whisker thalamocortical circuit. The neurons themselves were generic single compartment models. Given the reduced nature of the model, it is possible to analyze the model in detail. This analysis yields testable predictions. For example, the dependence of average spike rates of neuronal populations on the average spike rates of thalamic neurons (Fig 6b). These predictions could be tested in experiments where stimuli, applied for example with a magnetic stimulator, are applied during whisker movement at different amplitudes.

The main characteristics of the thalamocortical circuit are: (i) Strong external inputs to fast-spiking inhibitory neurons and excitatory neurons. (ii) Strong inhibition within L4, implementing feedforward and lateral inhibition. (iii) Recurrent excitation. iv) A brief but nonzero delay $\tau_{\text{delay}}^{\text{EI}}$ between the spike of an inhibitory neuron and the onset time of the inhibitory post-synaptic conductance in a post-synaptic neuron. Parameters based from neurophysiological measurements produce a model circuit with behavior that is in qualitative agreement with *in vivo* measurements over a large range of conditions. However, it was necessary to adjust the model parameters slightly (i.e. within a factor of 2–3; Methods) to best match experimental data (reference parameter set, Methods). Experimental inaccuracy, sampling errors, and differences across biological conditions (such as *in vitro* brain slices versus behaving brain) likely preclude more accurate estimation of synaptic parameters. Changing even a single parameter could lead to quantitative, and sometimes even qualitative changes in behavior (Figs 8–10), as has been noted in other contexts [75]. This means that the detailed parameters matter. For example, if *g*_II_ is too strong then L4I neurons do not respond sufficiently briskly to reduce whisking signals in L4E neurons (Fig 9*g* and 9*h*); on the other hand, if *g*_II_ is too weak then inhibition shuts down L4E neurons. Similarly, *g*_EE_ > 0 is required to amplify touch signals, but elevating *g*_EE_ by a factor of two beyond the optimal level causes run-away excitation (Fig 8*b*). Moreover, the kinetics matter, such as the durations of the I-to-E synaptic delay $\tau_{\text{delay}}^{\text{EI}}$ (Fig 7*a*), axonal delays, the time-courses of synaptic conductances (Fig 7*b*), and intrinsic neuronal properties.

### Comparison with previous L4 models

Rate models have been used to study cortical responses to fast-rising stimuli in the whisker system [8, 61] and elsewhere [71]. These previous models of L4 aimed to account for fast cortical responses to passive whisker deflections [8], but rate models cannot reliably describe brief responses to rapidly-varying stimuli [76]. Moreover, in important aspects these models have opposite behavior to our experimental and modeling results and to the known anatomy and physiology of L4 circuits. First, the modeled L4 neurons show activity in the absence of input, in contradiction to recent measurements [37, 77]. Second, L4 neurons exhibit strong and brief response to touch even for $\tau_{\text{delay}}^{\text{EI}}=0$, in contrast to our model.

The propagation of synchronous and brief activity in cortical circuits has been investigated [2, 63, 64, 78, 79]. In these models, excitatory inputs produce strong inhibition in local circuits, which is slightly delayed with respect to the excitation. In L4 of the barrel cortex, the convergence of thalamocortical input onto L4 FS neurons is higher than for L4 excitatory neurons (probabilities of connections are 0.75 vs 0.4) [34] and thalamic stimuli produce larger synaptic potentials in L4 FS than L4 excitatory neurons [35]. These features allow propagation of brief synchronous activity across network layers. These models can respond to brief, strong stimuli by brief responses of the L4 E and I neuronal populations. Our model goes beyond this effect by showing how this brief touch response can be obtained together with large *ν*_I_ and small *ν*_E_ in response to baseline and whisking. For example, simple feedforward models such as [35] cannot exhibit small *ν*_E_ for a wide range of *A*_T_, corresponding to both baseline and whisking. That model, however, explores the development of cortical responses of L4E and L4I neurons over the long time scale of short-term synaptic plasticity. Conducting such a study in a model with recurrent connections and comparing its outcome to in vivo measurements remains to be carried out.

Models of networks of strongly-coupled neurons compensated by inhibition have been studied extensively [38–40, 58, 80]. If inhibition on the excitatory neurons is not too large, the population-average response of excitatory and inhibitory neuron (*ν*_E_ and *ν*_I_) scales linearly with the external (here, thalamic) input *A*_T_ [39, 81]. The slope of the *ν*_E_ vs. *A*_T_ increases with *g*_II_ and decreases with *g*_IE_. If inhibition is too strong, *ν*_E_ = 0 and *ν*_I_ scales linearly with *A*_T_. In L4 and our model network, the numbers of neurons and the strengths of synaptic conductances are not very large. As a result, when *ν*_E_ is small (*ν_E_* ≲ 1 Hz), similar to measured values [36, 37], *ν*_E_ can (except of near firing threshold, namely the *A*_T_ values for which cortical neurons start firing) increase weakly with *A*_T_, be independent of *A*_T_, or even decrease with *A*_T_ (Figs 9*e*, 9*h*, 10*b* and 10*e*). Similar behavior was obtained in rate models of V1 [82, 83]. This result mimics the empirical observation that during transition from non-whisking to whisking, the spike rates of VPM neurons more than double, the spike rates of L4I neurons increase proportionally, but the spike rates of L4E neurons remains low, with no significant increase with thalamic input strength. For larger *ν*_E_ (> 2 Hz), *ν*_E_ depends linearly on *A*_T_. Larger *ν*_E_ could be obtained with larger *g*_II_ or smaller *g*_EI_ (Fig 9*b*, 9*e*, 9*h*), or by suppressing a fraction of L4I neurons using halorhodopsin activation (Fig 11*g*).

Based on the linearity of strongly-coupled networks, one would expect that if responses of L4E neurons to whisking is an order of magnitude smaller than that of VPM neurons, the same proportions will maintain for the responses to touch, whereas experimentally the touch responses of L4 excitatory and VPM neurons differ by only a factor of two. We explain this effect by the fact that, with $\tau_{\text{delay}}^{\text{EI}}$, inhibition lags excitation in response to brief and strong thalamic input. Indeed, for $\tau_{\text{delay}}^{\text{EI}}=0$, the response to L4E neurons is significantly reduced (Fig 7*a*).

In this paper we emphasize the suppression of tactile reafference signals in the somatosensory cortex. However, reafference signals could still contribute to cortical computation. Consistent with previous studies [16, 18, 84], we observed that spike rates were modulated by the phase of the whisking cycle [37]. Spike rates were larger for certain phases of the whisker motion, and individual neurons had different preferred phases. Neurons in VPM and L4 all showed modulation with whisking phase. Excitatory and inhibitory inputs to L4 excitatory neurons showed phase-tuning. Across the population, the phase-tuning of inhibition from L4 fast-spiking neurons is biased towards protractions. This suggests that the touch-evoked responses in L4 excitatory neurons may be modulated by whisking phase, as has been previously suggested [84]. This kind of phase-dependent modulation could play a role in localizing objects by whisker touch during active sensation.

## Materials and methods

### Experimental methods

The experimental methods were described in [37]. In brief, we performed chronic multi-electrode silicon probe recordings from VPM and cell-attached recordings from L4 excitatory and L4 fast spiking neurons. To search for VPM neurons we lightly anesthetized the mice (0.6–1.2% isoflurane) and stimulated individual whiskers during extracellular recordings. We mapped the principal whisker (PW) to assess whether the PW was one of the large whiskers that can be tracked reliably during behavior. We stimulated several individual whiskers around the PW with a piezoelectric stimulator at multiple frequencies (i.e. 5,10,20,40 Hz) and recorded the neural activity. Before and/or after each day of behavioral recording we confirmed that neurons respond to stimulation of the PW (we re-positioned the electrode drive daily). In addition, after placing the animal in the behavioral apparatus, we usually maintained the anesthesia for several minutes to check that neurons still responded to the PW (by manual stimulation and/or by contacting the whiskers with the pole). Animals performed the behavioral task shortly after anesthesia was withdrawn.

#### Statistical analyses

Data is presented as mean ± s.d. or mean ± s.e.m. as noted. Statistical comparisons use the non-parametric, two-sided Wilcoxon rank sum test. All statistical analyses were performed in Matlab (Mathworks).

#### Behavior, videography and recordings

In [37] we recorded from VPM neurons in mice trained to localize an object using their whiskers [48]. In each trial, during a sample epoch lasting a few seconds (1.1–2.5, mean 1.9 s), a pole appeared in one of multiple locations on the right side of the head (Fig 1a). Animals had to withhold licking when the pole was presented in the most anterior location (typically out of reach of the whiskers), or lick to obtain a reward when the pole was in the posterior locations. High-speed videography and automated whisker tracking quantified whisker movement (azimuthal angle, *θ*; whisking phase, ϕ), changes in curvature caused by the forces exerted by the pole on the whisker (change in curvature, Δκ)[36, 50, 85], and contact time, all with 1 millisecond temporal precision [49, 51]. Mice touched the pole multiple times (mean number of touches, 8.9±4.2 before reporting object location with licking (mean reaction time 416 ± 165 ms; mean ± s.d.). Recordings were made in trained mice (71±7% correct responses). To examine the relationship of VPM activity and tactile behavior we aligned spikes of individual neurons recorded in VPM with high-speed recordings of whisker movement and touch [36, 50, 51].

### Neuronal network model of a layer 4 barrel

Comprehensive neuroanatomical and neurophysiological data sets are revealing the connectivity between defined cell types over multiple spatial scales [28, 43, 44, 46, 86, 87]. But links between neural representations, computation and detailed anatomy are rarely achieved [6, 88]. One challenge is a lack of knowledge about strengths and dynamics of synapses between specific cell types during behavior. Most of such studies were carried out in slices, and there are differences between intrinsic and synaptic properties measured in slices and *in vivo* and between different *in vivo* states. Therefore, our strategy is to set parameter values (synaptic conductances, connectivity) close to measured *in vitro* values. However, differences between *in vitro* and *in vivo* conditions and experimental errors (e.g. estimates of unitary synaptic strengths) currently preclude exact quantification of the synaptic and connectivity properties (*e*.*g*., [53]). We define a set of parameter values, named "reference parameter set" [89], close to measured values when known (Fig 5), allowing for adjustment over a restricted range (mostly within a factor of 2 relative to empirical values), such that the circuit displays dynamics similar to experimentally-observed behavior. The reference parameter set specified below is used unless otherwise stated. Then, we vary one or two parameters to explore the role of those parameters on the system dynamics (*e*.*g*., Figs 8–10).

#### Network architecture

We modeled one barrel in layer 4 (L4) of the vibrissa primary somatosensory cortex (vS1). Our model circuit consists of L4 excitatory (E) neurons and L4 FS (I) neurons [28] receiving input from VPM (T) neurons (Fig 5a). When referring to modeling results we denote VPM neurons as T, L4 excitatory neurons as L4E, and GABAergic interneurons as L4I (Fig 5b and 5c). Non-FS interneurons have not been studied during behavior and are therefore not included in the model.

#### Dynamics of single cortical neurons

Intrinsic neuronal properties of L4 neurons have not been fully characterized. Therefore, following previous work on cortical dynamics [58, 59], single neurons are governed by a modified Wang-Buzsáki model with one compartment. The membrane potential $V_{i}^{\alpha }$, where α = E,I and *i* = 1,…, *N*_α_ obeys

$$C\frac{dV_{i}^{\alpha }}{dt}=-I_{\text{L},i}^{\alpha }-I_{\text{Na},i}^{\alpha }-I_{\text{Kdr},i}^{\alpha }-I_{\text{KZ},i}^{\alpha }-I_{\text{syn},i}^{\alpha }$$ (2)

where *C* is the neuron capacitance,

$$I_{\text{L},i}^{\alpha }=g_{\text{L}}^{\alpha }(V_{i}^{\alpha }-V_{L})$$ (3)

is the leak current, the conductance of the leak current is *g*_L_ = 0.05 mS/cm^2^ and 0.1 mS/cm^2^ for the excitatory and inhibitory neurons, respectively, and *V*_L_ = -65 mV. The current $I_{\text{syn,}i}^{\alpha }$ is the total synaptic input into the neuron. The other ionic currents are: $I_{\text{N}\text{a}}=g_{\text{N}\text{a}}m_{\infty }^{3}h(\text{V-}V_{\text{Na}})$ where *m*_∞_ = *α*_*m*_(V)/(*α*_*m*_(V)+*β*_*m*_(V)), *I*_Kdr_ = *g*_Kdr_ n^4^(V−*V*_K_), and *I*_KZ_ = *g*_KZ_ z(V-*V*_K_). The kinetics of *h* and *n* are given by:

$$dh/dt=\phi [\alpha_{h}(V)(1-h)-\beta_{h}(V)h],\;\;dn/dt=\phi [\alpha_{n}(V)(1-n)-\beta_{n}(V)n]$$ (4)

The functions *α*(V) and *β*(V), for *V* in mV, are:

$$\alpha_{m}(V)=0.1(V+30)/\{1-\text{exp}[-0.1(\text{V}+30)]\}$$ (5)

$$\beta_{m}(V)=4\text{exp}[-(\text{V}+55)/18]$$ (6)

$$\alpha_{h}(V)=0.7\text{exp}[-(V+44)/20)]$$ (7)

$$\beta_{h}(V)=10/\{1+\text{exp}[-0.1(V+14)]\}$$ (8)

$$\alpha_{n}(V)=0.1(V+34)/\{1-\text{exp}[-0.1(\text{V}+34)]\}$$ (9)

$$\beta_{n}(V)=1.25\text{exp}[-(V+44)/80].$$ (10)

We set *ϕ* = 0.2 to produce wider spikes than in [59]. The gating variable z of the adaptation current *I*_KZ_ is governed by the equation:

$$dz/dt=[z_{\infty }(V)-z]/\tau_{z}$$ (11)

Where *z*_∞_(V) = 1/{1+exp[-0.7(V+30)]} and τ_z_ = 60 ms. The parameters of the model are: *g*_Na_ = 100 mS/cm^2^, *V*_Na_ = 55 mV, *g*_Kdr_ = 40 mS/cm^2^, *V*_K_ = -90 mV, *C* = 1μF/cm^2^. Excitatory neurons have *g*_KZ_ = 0.5 mS/cm^2^, whereas inhibitory neurons do not possess this current (*g*_KZ_ = 0).

#### Network architecture

The network consists of *N*_E_ excitatory neurons and *N*_I_ inhibitory neurons. The L4 neurons receive input from *N*_T_ thalamic relay neurons in one barreloid. For the simulations, *N*_E_ = 1600, *N*_I_ = 150, and *N*_T_ = 200 [28]. Each neuron, whether excitatory or inhibitory, receives excitation and inhibition from the excitatory and inhibitory cortical neuronal populations respectively, as well as excitation from VPM thalamic neurons. The probability that a neuron from the *β*th, pre-synaptic population forms a synapse on a neuron from the *α*th, post-synaptic population is *K*_*αβ*_/*N*_*β*_ [90]. Therefore, a neuron from the *α*th population receives, on average, *K*_αβ_ synaptic inputs from neurons in the *β*th population. We define the matrix $C_{ij}^{\alpha \beta }$ to be 1 if the *j*th neuron from the *β*th population projects to the *i*th neuron from the *α*th population, and 0 otherwise.

#### Synaptic input

We consider only fast synapses corresponding to AMPA and GABA_A_ receptors. The total synaptic input a cell *i* from population *α* receives is the sum of the synaptic currents from all the pre-synaptic populations:

$$I_{\text{syn},i}^{\alpha }=\sum_{\beta =\text{T},\text{E},\text{I}}G_{\text{syn},i}^{\alpha \beta }(t)\left(V_{i}^{\alpha }-V_{\text{syn}}^{\beta }\right)$$ (12)

The total conductance $G_{\text{syn},i}^{\alpha ,\beta }$ for *β* = T,E,I is:

$$G_{\text{syn},i}^{\alpha \beta }(t)=\frac{\tau_{\text{syn},\text{all}}}{\sqrt{K_{\alpha \beta }}}g_{\alpha \beta }\sum_{j=1}^{N}C_{ij}^{\alpha \beta }s_{{}_{j}}^{\alpha \beta }(t)$$ (13)

where the normalization constant τ_syn,all_ is set to be 1 ms. The value $s_{j}^{\alpha \beta }$ denotes a synaptic variable of the pre-synaptic neuron *j* from the *β*th population projecting to the *α*th population. Those variables for AMPA and GABA_A_ conductances are:

$$s_{j}^{\alpha \beta }(t)=\frac{1}{t_{\text{syn}}}\sum_{k}\text{exp}\left[-(t-t_{k}^{\beta }-\tau_{\text{delay}}^{\alpha \beta })/t_{\text{syn}}\right]\Theta (t-t_{k}^{\beta }-\tau_{\text{delay}}^{\alpha \beta })$$ (14)

The pre-synaptic neuron fires at times $t_{k}^{\beta }$, *k* is the spike index, *t*_syn_ is the time constant of synaptic decay, Θ is the Heaviside function, and $\tau_{\text{delay}}^{\alpha \beta }$ is the time delay between the firing by a spike from a presynaptic neuron from the *β*th population and the onset of the post-synaptic conductance in the post-synaptic neuron from the *α*th population. Unless specified otherwise, we use time constants *t*_syn_ = *t*_AMPA_ = 2 ms for AMPA-mediated synapses and *t*_syn_ = *t*_GABAA_ = 3 ms for GABA_A_-mediated synapses. The reversal potentials are V_AMPA_ = 0 and V_GABAA_ = -85 mV. The values of *g*_*αβ*_, *K*_*αβ*_ and $\tau_{\text{delay}}^{\alpha \beta }$ are based on [27, 28, 34, 53, 56, 60] (Table 2). We scale the conductance strength like $1/\sqrt{K_{\alpha \beta }}$ (Eq 13) to allow comparison of our conductance values *g*_*αβ*_ with previous publications [40, 58], but analyze the circuits only for *K*_*αβ*_ values of corresponding to those of mouse L4 barrel and do not investigate the circuit dynamics as values of *K*_*αβ*_ vary. Compared to literature values the *K*_αβ_ were reduced by a factor of 2 for the following reasons. First, this reduction counteracts strong synchrony in the cortical circuits. Second, this could have been achieved by modeling highly variable synaptic strengths [91]. Third, L2/3 connectivity has a long tail of strong connections [92]. Similar heterogeneity in connections may appear also in L4. The heterogeneity increases the effective sparseness of the synaptic connection matrix. Assuming lower values of *K*_αβ_ is like taking into account strong connections only, including those in the tail of the distribution. The effect of this manipulation on the total synaptic conductance a neuron receives is within the factor 2–3 of resemblance between the experimentally-measured values (in slice experiments) and the values used in the model (Table 2).

The experimental evolution of the response of VPM and L4 neurons to consecutive touch events has not reported yet. In this work, we do not address how the responses to whisking and touch vary over the long time scale of short-term plasticity. For these reasons, and to keep the model and its analysis simple, the synapses in the model do not have depression and facilitation properties.

#### Spike rates of thalamic neurons

Spike trains of T neurons were modeled as statistically-independent inhomogeneous Poisson processes. All T neuron share the generating function *F*_T_ mimicking thalamic activity during quiescence, whisking or whisking and touch (Fig 5*d*):

$$F_{\text{T}}(t)=A_{\text{T}}\left[1+B_{\text{T}}\text{sin}\left(\frac{2\pi t}{\tau_{w}}+\phi \right)\right]+\frac{C_{\text{T}}}{\tau_{c}}\Theta (t-n\tau_{w}-t_{c})\Theta (n\tau_{w}+t_{c}+\tau_{c}-t)$$ (15)

The first term in Eq 1 represents the baseline and whisking modulation contribution to the instantaneous spike rate, and the second term represents spikes added by touch. The parameter *A*_T_ is the spike rate averaged over a whisker movement cycle without touch, *B*_T_ is the modulation depth, and *C*_T_ is the average number of additional spikes per touch, *τ*_*w*_ is the whisking period, *t*_c_ is the time of touch onset within a whisking cycle, and *n* is the cycle number. During whisker movements, *A*_T_ increases above a baseline, with sinusoidal modulation phase-locked to a single preferred phase *ϕ* (several neurons in VPM exhibit phase-locking activity to the whisking cycle;[37]). Touch is represented by adding a rectangular function at touch onset *t* = *t*_c_ (with respect to the whisking cycle), stretched over 3 ms with an integral of *C*_T_ = 0.6 spikes per touch (Eqs 1 and 15). We use the parameters: *B*_T_ = 0.25 (based on the data of Supplementary Fig. 2d in [37]), *τ*_*c*_ = 3 ms, *T* = 100 ms, *ϕ* = π/2. The parameter *t*_c_ is set to be 0.5*T* because thalamic neurons reach their maximal spike rate during whisking at around maximal retraction [37]. The parameters *A*_T_ and *C*_T_ are 6 Hz and 0 during no-whisking states, 14 Hz and 0 during whisking states and 14 Hz and 0.6 during whisking-and-touch states. For simplicity, we take the same values of *A*_T_, *B*_T_ and *C*_T_ for all thalamic neurons. Because of the large convergence of thalamic inputs into L4E and L4I neurons, our results are not modified significantly if we allow those thalamic parameters to be taken from a uniform distribution with the same average values.

#### Population- and time-averaged quantities

We define *ν*_*α*_ to be the population- and time-averaged spike rates of all the neurons in the *α*th population over many whisking cycles. Touch response of a neuron is defined to be the difference between the number of spikes fired by that neuron during a time window of 25 ms after touch onset and the number of spikes fired by the neuron 25 ms before touch onset. We define *R*_*α*_ to be the population- and time-average of the spiking response to touch of all the neurons in the *α*th population over many touch events.

#### Simulations of halorhodopsin activation

We model activation of halorhodopsin, a light-gated chloride pump, expressed in L4I FS neurons (Figs 11 and 12). Halorhodopsin expression levels likely varied widely across individual neurons [65]. We therefore assume that halorhodopsin is expressed in only a fraction *f*_halo_ of the inhibitory neurons, 0 ≤ *f*_halo_ ≤ 1. Activation by light of the *i*th FS neurons that express halorhodopsin was modeled by two processes. First, as a chloride pump, its activation has an effect of negative current injection to the neuron. The amplitude of this current in the *i*th neuron is *I*_halo,i_ = *I*_halo,0_ + *I*_halo,1_*x*_*i*_, where *x*_*i*_ is taken randomly from a uniform distribution between -1 and 1, *I*_halo,0_ = -2 μA/cm^2^, and *I*_halo,1_ = 1 μA/cm^2^. As a result, *I*_*halo*,*i*_ varies between -3 μA/cm^2^ and -1 μA/cm^2^. Second, the GABA_A_ reversal potential is depolarized [93, 94] by a value Δ*V*_GABAA,*i*_ = *βI*_halo,*i*_, where *β* = -4mVcm^2^/μA. The second process has only a small quantitative effect on the spiking responses of cortical neurons.

#### Numerical methods

Simulations were performed using the fourth-order Runge-Kutta method with time step Δ*t* = 0.05 ms. Simulations with smaller Δ*t* reveal similar statistics of neuronal firing patterns, such as spike rates ν (averaged over many whisking cycles), touch responses *R* or levels of synchrony. Differences between individual voltage time courses, however, diverged over large integration time interval. This divergence is expected because of the chaotic nature of the sparse network dynamics [38, 39, 95]. Statistics were computed after removing a transient of 0.5 s, over 60 s for panels showing dynamical properties of one realization (Figs 6*a*, 6*c*, 6*d*, 8*d*–8*i* and 11*a*–11*f*) and over 5.5 s for panel showing dependence on parameters (Figs 6*b*, 7, 8*b*, 8*c*, 9, 10, 11*g*, 11*h* and 12) where each data point is computed by averaging over 10 network realizations.
